# High SHBG and Low Bioavailable Testosterone are Strongly Causally Associated with Increased Forearm Fracture Risk in Women: An MR Study Leveraging Novel Female-Specific Data

**DOI:** 10.1007/s00223-024-01301-5

**Published:** 2024-10-16

**Authors:** Johan Quester, Maria Nethander, Eivind Coward, Ene Reimann, Reedik Mägi, Estonian Biobank Research Team, Andres Metspalu, Lili Milani, Tõnu Esko, Reedik Mägi, Mari Nelis, Georgi Hudjashov, Ulrika Pettersson-Kymmer, Kristian Hveem, Claes Ohlsson

**Affiliations:** 1 Department of Internal Medicine and Clinical Nutrition, Institute of Medicine, Sahlgrenska Osteoporosis Centre, Centre for Bone and Arthritis Research at the Sahlgrenska Academy, University of Gothenburg, Gothenburg, Sweden; 2 Department of Drug Treatment, Region Västra Götaland, Sahlgrenska University Hospital, Gothenburg, Sweden; 3 Bioinformatics Core Facility, Sahlgrenska Academy, University of Gothenburg, Gothenburg, Sweden; 4 HUNT Center for Molecular and Clinical Epidemiology, Department of Public Health and Nursing, NTNU, Norwegian University of Science and Technology, 7491 Trondheim, Norway; 5 Estonian Genome Center, Institute of Genomics, University of Tartu, Tartu, Estonia; 6 Department of Medical and Translational Biology, Clinical Pharmacology, Umea University, Umea, Sweden; 7 HUNT Research Centre, Department of Public Health and Nursing, Norwegian University of Science and Technology, Postboks 8905, 7491 Trondheim, Norway; 8 Levanger Hospital, Nord-Trøndelag Hospital Trust, Levanger, Norway

**Keywords:** Mendelian randomization analysis, Female, Fractures, Bone, Sex hormone-binding globulin, Testosterone, Bone density

## Abstract

The effects of androgens on women’s bone health are not fully understood. Mendelian randomization (MR) studies using sex-combined data suggest that sex hormone-binding globulin (SHBG) and bioavailable testosterone (BioT) causally affect bone traits. Given significant sex differences in hormone regulation and effects, female-specific MR studies are necessary. In the current study, we explored the causal relationships of SHBG, BioT, and total testosterone (TT) with forearm fracture (FAFx) risk in women using two-sample MR analyses. We utilized a unique female-specific FAFx outcome dataset from three European biobanks (UFO, HUNT, Estonian Biobank) comprising 111,351 women and 8823 FAFx cases, along with female-specific genetic instruments of SHBG, BioT, and TT identified in the UK Biobank. We also assessed bone mineral density (BMD) at the forearm (FA), femoral neck (FN), and lumbar spine (LS) using female-specific GWAS data from the GEFOS consortium. High SHBG (odds ratio per standard deviation increase (OR/SD): 1.53, 95% confidence intervals (CIs): 1.34–1.75), low BioT (OR/SD: 0.77, 0.71–0.84) and low TT (OR/SD 0.90, 0.83–0.98) were causally associated with increased FAFx risk. BioT was positively, and SHBG inversely, causally associated with especially FA-BMD, but also LS-BMD and FN-BMD, while TT was only significantly positively associated with FA-BMD and LS-BMD. We propose that endogenous androgens and SHBG are important for women’s bone health at distal trabecular-rich bone sites such as the distal forearm and may serve as predictors for FAFx risk.

**Supplementary Information:**

The online version contains supplementary material available at 10.1007/s00223-024-01301-5.

## Introduction

It is estimated that one-third of postmenopausal women in Europe will sustain a fragility fracture during their remaining lifetime, a risk on par with that of cardiovascular disease [1]. The economic impact of osteoporotic fractures is substantial, with associated costs being up to six times higher than those for patients without fractures in the US population [2].

The beneficial effects of estrogens on bone disease in women are well established [3]. The effects of androgens, however, remain less well understood. Androgens can influence bone directly through their interaction with androgen receptors, or indirectly by binding to estrogen receptors α and β following aromatization in fat or other tissues [4]. Preclinical data from studies involving female mice devoid of androgen receptors and ovariectomized rats treated with dihydrotestosterone (DHT), a non-aromatizable androgen, suggest that androgen receptor signaling is of importance for maintaining the health of trabecular bone in females [5–7]. Interestingly, in women diagnosed with complete androgen insensitivity syndrome (CAIS)—a condition caused by a loss of function mutation in the gene encoding the androgen receptor—decreased bone mineral density (BMD) is more commonly observed at the trabecular-rich lumbar spine than the femoral neck, which mainly consists of cortical bone [8, 9].

Only a few percent of circulating testosterone (T) circulates freely, with the majority bound to and transported by sex hormone-binding globulin (SHBG) and albumin [10]. The free hormone hypothesis, though still somewhat controversial, states that it is mostly the free hormone concentration that accounts for the hormone’s biological activity and thus best correlates with any biological effects. Although several observational studies have demonstrated that low levels of circulating SHBG [11–16] or high levels of circulating free T (or bioavailable T, BioT) [11, 13, 17–22] are associated with bone health parameters in women, results are far from consistent [12, 18, 23–27]. As for circulating total T (TT), the findings are even less compelling [13, 15, 17, 18, 23, 25, 28–31], with only a handful of observational studies indicating a relationship [11, 21, 32].

Thus, while preclinical findings suggest that androgens play a protective role in bone health, the effects of androgens on bone health in women still remain unclear as discrepancies of observational studies could be explained by biases, such as residual confounding (e.g., age, comorbidities and medication) and reverse causality. Mendelian randomization (MR) can overcome several shortcomings in observational studies by combining genetic information fixed at birth (eliminating reverse causation bias) and observational data to create possible unbiased causal associations between an exposure and outcome [33]. Because germline genetic variants are selected at random during meiosis, MR is less likely to be influenced by environmental and lifestyle factors.

Recently, a few sex-combined MR studies have suggested that SHBG or BioT may causally affect BMD [34–36], osteoporosis risk [37, 38], or fracture risk [39, 40]. However, as the regulation of circulating levels of these androgen-related hormones (exposures in MR) and their effects (outcomes in MR) differ substantially between men and women, sometimes showing opposing effects between sexes [41], it is essential to conduct female-specific MR analyses to understand the effects of androgens on bone traits in women. Such analyses require both female-specific genetic instruments and availability of female-specific genome-wide association study (GWAS) summary data for the outcomes being investigated in the MR.

In the present study, our aim was to use a two-sample MR framework to specifically explore the causal relationship between female androgen levels and bone health in women. As instruments we employed strong female-specific genetic signals for circulating SHBG, BioT, and Total Testosterone (TT) from the UK Biobank [41]. These signals were combined with a large and unique collection of female forearm fracture cases derived from three Nordic biobanks. Forearm fractures are common, occur relatively early in life, and show high heritability, providing increased statistical power and more precise estimates compared to other osteoporotic fractures [42]. Additionally, as a secondary aim, we assessed the causal association between SHBG, BioT, and TT and female-specific measures of femoral neck BMD, lumbar spine BMD, and forearm BMD. For sensitivity analyses, we further refined our MR analyses by focusing exclusively on cis single-nucleotide polymorphisms (SNPs) related to SHBG. Cis-SNPs, which are positioned close to or within the gene encoding the protein in question, are thought to offer a more accurate assessment of the causal role of a circulating protein in relation to a disease or trait [43]. Restricting an MR analysis to cis-SNPs thus reduces the risk of horizontal pleiotropy, where a genetic variant affects multiple unrelated traits through different biological pathways, compared to using trans-SNPs that are located farther from the gene and therefore are more likely to exert broader, unrelated effects.

## Methods

### Study Design

We performed a two-sample MR study using only female exposure and outcome data to assess the causal association of TT, SHBG, and BioT (exposures) with forearm fracture risk (primary outcome) and BMD (secondary outcome). As instrumental variables, we used female-specific SNPs from the largest available GWAS of circulating levels of TT, SHBG, and BioT in women [41]. As outcome measures, we used female-specific case–control data of forearm fractures collected from four large biobanks (*Estonian Biobank* from Estonia, *HUNT* from Norway, *UFO* from Sweden, and *UK Biobank* from the United Kingdom), and female-specific DXA-derived BMD GWAS summary statistics available from the GEFOS consortium (Table 1) [44, 45]. For a flowchart of the study methodology, see Supplementary Fig. 1. The current MR study relies on three core assumptions: The genetic instruments (1) are associated with the exposure of interest (relevance), (2) are not associated with confounders of the association between the exposure and the outcome (independence), and (3) only influence the outcome through the exposure and not directly or through alternative (horizontally pleiotropic) pathways (exclusion restriction). The paper is reported on the basis of recommendations by the Strengthening the Reporting of Observational Studies in Epidemiology Using Mendelian Randomization Guidelines (STROBE-MR) [46].

**Table 1:** Description of female-specific data used for each phenotype

Variable	Exposure/Outcome	Source	Ancestry	Sample size	Cases
**Sex hormones**					
SHBG	Exposure	UKBB, Ruth et al., PMID: 32,042,192	EUR	189,473	-
Bioavailable testosterone	Exposure	UKBB, Ruth et al., PMID: 32,042,192	EUR	188,507	-
Total testosterone	Exposure	UKBB, Ruth et al., PMID: 32,042,192	EUR	230,454	-
**Fractures**					
Forearm fractures	Outcome, one-sample MR	UKBB	EUR	237,572	11,564
Forearm fractures	Outcome, two-sample MR	EstBB; HUNT; UFO	EUR	111,351	8823
Forearm fractures	Outcome, overlap-sample MR	UKBB; EstBB; HUNT; UFO	EUR	348,923	20,387
**BMD**					
Femoral neck BMD	Outcome, two-sample MR	GEFOS, Estrada et al., PMID: 22,504,420	Mainly EUR	22,900	-
Lumbar spine BMD	Outcome, two-sample MR	GEFOS, Estrada et al., PMID: 22,504,420	Mainly EUR	22,177	-
Forearm BMD	Outcome, two-sample MR	GEFOS, Zheng et al., PMID: 26,367,794	EUR	7848	-

*EstBB* Estonian Biobank, *HUNT* The Trøndelag Health Study, *UFO* Umeå Fracture and Osteoporosis Study, *UKBB* UK Biobank, *GEFOS* Genetic Factors for Osteoporosis consortium, *MR* Mendelian randomization, *BMD* Bone mineral density, *EUR* European ancestry

### Exposures

#### Data Source and Population

We obtained genetic predictors for the exposures from the largest available female-specific GWAS meta-analysis of TT, SHBG, and BioT [41]. This GWAS used data from the UK Biobank, a large, ongoing, prospective cohort study that comprises over 500000 participants, recruited from 2006 to 2010 across the United Kingdom at the age of 37 to 73 years [47]. Participants who self-reported by questionnaire as being of an ancestry other than white European were excluded in the GWAS as well as women who self-reported taking hormone-based medication at the time of the initial visit [41].

#### Measurements of TT, SHBG and BioT

SHBG and TT were measured in nmol/L by chemiluminescent immunoassay analyses on a Beckman Coulter DXI 800 [48]. BioT was calculated from TT, SHBG, and albumin (measured in g/L by a colorimetric analysis on a Beckman Coulter AU5800) using the Vermeulen equation [41]. The regression model for the effect sizes of the genetic variants on the exposures included several covariates: All models for TT, SHBG, and BioT included genotyping chip, age at baseline, and ten genetically derived principal components. TT also included fasting time and assessment center as covariates. SHBG and BioT also included batch, dilution, menopause status, operation status, minutes since blood draw, and time of blood draw. Additionally, the main SHBG model included body mass index (BMI) to increase statistical power by reducing trait variance [41]. Similarly, as suggested by Ruth et al., we used genetic variants from the BMI-adjusted analyses but applied effect estimates from BMI-unadjusted analyses [41]. TT and SHBG were transformed using rank-based inverse normal transformation, whereas BioT was transformed using the natural log. To ease interpretation, we estimated standardized effect estimates for natural log BioT using available natural log effect estimates and standard errors, allele frequencies, and number of women as described by Rietveld et al. [49].

#### Genetic Predictors of TT, SHBG, and BioT

The female-specific GWAS revealed 254 independent (*r*^2^ < 0.05) genome-wide significant (*P*-value < 5 × 10^–8^) genetic instruments for TT (*N* = 230,454), 359 independent instruments for SHBG (*N* = 189,473), and 180 independent instruments for BioT (*N* = 188,507) [41]. All genetic variants had an imputation quality score > 0.5 and minor allele frequency > 0.1%. The genetic correlation between SHBG and TT was weak (*r*_g_ =  − 0.06), but there was a strong negative correlation between BioT and SHBG (*r*_g_ =  − 0.74) and a similar positive correlation between TT and BioT (*r*_g_ = 0.65). Quantifying instrument strength using the t-statistic approximation of the F-statistic, each genetic instrument showed an F-statistic greater than 10, indicating strong genetic instruments. Harmonization with the outcome data was performed using the TwoSampleMR package, with attempt to infer positive strand alleles using allele frequencies for palindromes. In case genetic instruments were missing in the outcome data, suggested proxies in the supplement of Ruth et al. were used [41]. As cis-SNPs for SHBG, we selected three commonly occurring variants utilized in previous studies: rs6761, rs1799941, and rs858519 [41, 50, 51]. For outcomes restricted to HapMap variants, we used rs12150660 as a proxy for rs1799941 and rs727428 as a proxy for rs858519.

### Outcomes

#### Forearm Fractures

For the two-sample MR analyses of forearm fractures, we utilized summary statistics derived from individual-level outcome data sourced from three large Northern European biobanks (Estonian Biobank from Estonia, HUNT from Norway, and UFO from Sweden). We also performed one-sample MR analyses using summary statistics calculated from individual-level data from the UK Biobank. In addition, we performed combined one-and two-sample MR using data from all four cohorts (overlap-sample MR). All four contributing biobanks obtained written informed consent from all participants and received approval from their respective research ethics committees. The UK Biobank has ethical approval from the Northwest Multicentre Research Ethics Committee (11/NW/0382). The UFO study was approved by the local research ethics committee at Umeå University (Umu dnr 03-426; EPN 2012-254-32 M; 2011/32-32 M; 2011-251-32 M). The HUNT study has been approved by the Regional Ethics Committee for Medical Research in Norway (REK 2015/615; REK 2014/144). Estonian Biobank has ethical approval from the Estonian Committee on Bioethics and Human Research at the Ministry of Social Affairs (No 1.1-12/624). For detailed information on the four contributing biobanks, please see Supplementary Table S1. Forearm fracture cases were identified by International Classification of Diseases (ICD) codes (ICD10, S52; ICD9, 813), for UK Biobank in combination with self-reported data. No self-reported forearm fracture data were used in Estonian Biobank, HUNT, or UFO (Table 2). Genotyping was performed in each cohort using Illumina genome-wide genotyping chips or in the case of UK Biobank, the UK Biobank Axiom Array, or the UK BiLEVE Array. Imputation was performed to ensure accurate ascertainment of common genetic variations above a minor allele frequency threshold of 1%. Logistic models, using the SAIGE (UK Biobank, Estonian Biobank, HUNT) or PLINK (UFO) software, were used to estimate the SNP association with fracture in each cohort, adjusting for age (simple and quadratic terms), height, weight, principal components, study site (when necessary), and family structure (if feasible). Menopausal status at the time of forearm fracture was not available for participants in some of the included cohorts, preventing us from adjusting or stratifying the analyses by menopausal status. However, the median age at fracture varied between 58 and 64 years in the included cohorts (Table 2), suggesting that most of the forearm fractures occurred after menopause. The effect estimates from the individual cohorts were then combined using fixed-effect inverse variance weighted meta-analysis. In the analyses, we only included female subjects of European descent with available data on genotypes and relevant covariates. For the cohorts from Estonian Biobank, HUNT, and UFO, individual participant-level data were available for in total 111 351 women, including 8 823 forearm fracture cases. For UK Biobank, data were available for 237 572 women, including 11 564 forearm fractures.

**Table 2:** Fracture details for individual forearm fracture studies

Study	Fracture assessment method	Fracture cases (*n*)	Controls (*n*)	All (*n*)	Median age at fracture (yrs)
UKBB	Medical records* and self-reported	11,564	226,008	237,572	60.5
UFO	Medical records* and radiographic verification	850	856	1706	61.4
HUNT	Medical records*	4447	31,581	36,028	64
EstBB	Medical records*	3526	70,091	73,617	58
Total		20,387	328,536	348,923	

*EstBB* Estonian Biobank, *HUNT* The Trøndelag Health Study, *UFO* Umeå Fracture and Osteoporosis Study, *UKBB* UK Biobank

*ICD codes: ICD10, S52; ICD9, 813

#### Lumbar Spine BMD and Femoral Neck BMD

Effect estimates for the associations between the genetic instruments and lumbar spine BMD (LS-BMD; *n* = 22 177) and femoral neck BMD (FN-BMD; *n* = 22 900) were derived from female-specific GWAS summary statistics available from the GEnetic Factors for OSteoporosis (GEFOS) consortium (www.gefos.org) [44]. These data originate from a meta-analysis of 17 populations across North America, Europe, East Asia, and Australia with a variety of epidemiological designs and subject characteristics. The mean age of women across the cohorts varied between 33 and 76 years with an overall mean age of 59 years. Genotyping was performed by each individual study following standard protocols, and imputation was then carried out on ~ 2.5 million single-nucleotide polymorphisms (SNPs) from HapMap Phase 2 release 22. BMD of lumbar spine (L1-4) (LS) and femoral neck (FN) was measured by Dual-energy X-ray absorptiometry (DXA) [44]. The effect of each gene variant on standardized BMD was adjusted for age, weight, and four principal components. As female-specific beta values and standard errors are not given for all SNPs in this publicly available dataset, we estimated these parameters from *P*-values, allele frequencies, and number of women as described by Rietveld et al. [49].

#### Forearm BMD

For effect estimates of associations between genetic instruments and forearm BMD (FA-BMD; n = 7 848), we used non-sex-specific GWAS summary statistics available from the GEFOS consortium (www.gefos.org) [45]. Although the dataset included a low number of male subjects, the female contribution was as high as 96%, and we thus consider these summary statistics as female-specific in our analyses. The FA-BMD data originate from a meta-analysis of five populations across North America, Europe, and Australia. The mean age of women across the cohorts varied between 48 and 71 years with an overall mean age of 61 years. Genotyping was performed by each individual cohort following standard protocols and the data were meta-analyzed using the software GWAMA [45]. BMD was measured using DXA. The effect of each gene variant was adjusted for sex, age, age^2^, weight, and standardized to have a mean of zero and a standard deviation of one.

### Statistical Analysis

#### Causal Effects

The primary MR analyses were performed using a two-sample summary statistics MR approach, where we obtained SNP-specific Wald estimates and then meta-analyzed them using inverse variance weighting (IVW) with random effects (RE), thereby estimating the total effect of the exposures (BioT, SHBG, and TT) on forearm fracture risk (using data from the Estonian Biobank, HUNT, and UFO), LS-BMD, FN-BMD, and FA-BMD. IVW-RE lessens the reliance of core MR assumption number three, that genetic variants should not act through additional parallel biological pathways (horizontal pleiotropy) and relies on the assumptions that pleiotropic effects among SNPs are random (balanced pleiotropy) and that their magnitude is independent of the magnitude of the corresponding genetic-exposure (G-X) effects, known as the InSIDE assumption [52]. In addition to the two-sample MR framework in the analyses of forearm fractures, we also included one-sample MR analyses using data from only the UK biobank, as well as an overlap-sample MR using outcome data from all four cohorts, i.e., Estonian Biobank, HUNT, UFO, and UK Biobank.

We also performed several sensitivity analyses using MR methods even less sensitive to horizontal pleiotropy. In these sensitivity analyses, we used (1) the weighted median method, which provides a causal estimate if at least 50% of the weight in the analysis is derived from valid instrumental variables [53]; (2) a mode-based estimation method, which assumes that the largest weighted contribution of “similar” (i.e., identical in infinite samples) SNP-specific MR estimates comes from valid instruments, thereby providing natural robustness against outlier variants (and hence likely are pleiotropic) [54]; (3) MR Egger method, which can detect and adjust for a non-zero average pleiotropic effect under the assumption that the instrument strength is independent of direct (pleiotropic) effects (InSIDE assumption) [55]; (4) MR-pleiotropy residual sum and outlier (MR-PRESSO) method, which uses a leave-one-out approach to detect pleiotropy and estimates causality after removing invalid IVs [56]; (5) MR-Lasso method, which models pleiotropic effects and then removes variants with non-zero pleiotropic effects [57]; and (6) MR-Robust Adjusted Profile Score (RAPS) that penalizes genetic variants that are pleiotropic and is robust to systematic and idiosyncratic pleiotropy. We also performed analyses after Steiger filtering, removing genetic variants that demonstrate higher correlation with the outcome than the exposure, thus filtering instruments that are likely to be arising due to reverse cause or potentially through horizontal pleiotropy [58, 59] (Supplementary Tables S3 and S4).

To ensure the robustness of our estimated associations, we performed additional sensitivity analyses by excluding SNPs associated with potential confounders. Confounders were identified based on variables from the FRAX® fracture risk assessment tool, including smoking, alcohol consumption, glucocorticoid use, rheumatoid arthritis, and body mass index (BMI) [60]. We also considered estradiol levels, physical activity, grip strength, educational attainment, and household income as confounders, acknowledging that some might also act as mediators in the exposure-outcome relationship. Any SNPs significantly associated with these confounders were excluded if they reached the Bonferroni-corrected P-value threshold of 7 × 10^–5^ (0.05/732, adjusted for the 732 unique exposure SNPs). We assessed the associations of the exposure SNPs with potential confounders using GWAS sourced from The MRC IEU OpenGWAS data infrastructure [61]. For a detailed list of the specific GWAS referenced, see Supplemental Table S6.

To assess heterogeneity between the instrumental variable estimates of different SNPs, we visualized the data with funnel and scatter plots [62]. Funnel plots (individual instrumental variable estimates plotted against the standard errors of the instrumental variable estimate) allow for assessment of unbalanced directional pleiotropy, any asymmetry in the funnel plot is a sign of directional pleiotropy and could lead to bias in the results. Scatter plots (genetic associations with the outcome plotted against the genetic associations with the risk factor) highlight the heterogeneity of the Wald ratios. If all variants are valid IVs, a dose–response relationship should be observed in the scatterplot, and if a majority of variants are valid IVs, pleiotropic variants may be detected. In addition to visual assessments, we analyzed the heterogeneity in the MR estimates across the genetic variants using the I2 index and the Q heterogeneity test [52].

For SHBG, we investigated the associations of cis-SNPs with all examined outcomes. Utilizing a cis-SNP design is thought to reduce the risk of confounding effects caused by pleiotropy [43]. We employed Wald ratios to test individual associations of each cis-SNP with forearm fracture and BMD. Due to the inclusion of only HapMap variants in the LS-BMD and FN-BMD data, rs6761 was the only available cis-SNP, thus requiring us to use proxy SNPs for rs1799941 and rs858519. We also performed combined analyses of all three SHBG cis-SNPs using IVW. Given a moderate correlation among the cis-SNPs (linkage disequilibrium R^2^ between 0.35 and 0.53), we applied a fixed-effect IVW method that accounts for this correlation [63].

All statistical analyses were performed using the software R (www.R-project.org) version 4.2.2 using the TwoSampleMR [64] and the MendelianRandomization [65] packages.

#### Power Calculations

We performed power calculations for the two-sample MR analyses of forearm fractures based on R code from an online power calculation tool (https://github.com/kn3in/mRnd/) [66]. The main determinants of power estimation in MR analysis are the sample size, the genetic variance explained by the instruments, and the size of the causal effect [66]. As the GWAS of TT, SHBG, and BioT did not provide information on the genetic variance explained by each SNP on TT, SHBG, or BioT, we estimated this measure using formula derived by Shim et al. [67]. With this approximation, the 180 SNPs associated with BioT explain 7.0% of the variance of BioT, the 359 SNPs associated with SHBG explain 17,6% of the variance, and the 254 SNPs associated with TT explain 9.3% of the variance. For forearm fractures, using an available sample size of 111 351 and 8 823 cases, we had 80% power to detect a causal association of odds ratio (OR) 0.89 per standard deviation (SD) increase in BioT, OR 1.07 per SD increase in SHBG, and OR 0.91 per SD increase in TT (Supplementary Fig. S2).

## Results

The median age at the time of fracture was consistent across all four female forearm fracture cohorts, ranging between 58 and 64 years (Table 2). Baseline characteristics, such as weight and height, were also comparable among the fracture cohorts (Supplementary Table S2). Both exposure and outcome data included only women, were predominantly derived from population-based studies of European descent, and involved participants within a similar age bracket. In our primary analyses, there was no overlap between the participants in the exposure and outcome datasets. These characteristics collectively reduce the likelihood of sample differences in pleiotropic pathways or linkage disequilibrium patterns and reinforce the validity of our causal inferences.

### Effect of TT, bioT, and SHBG on Forearm Fractures

**Fig. 1:**
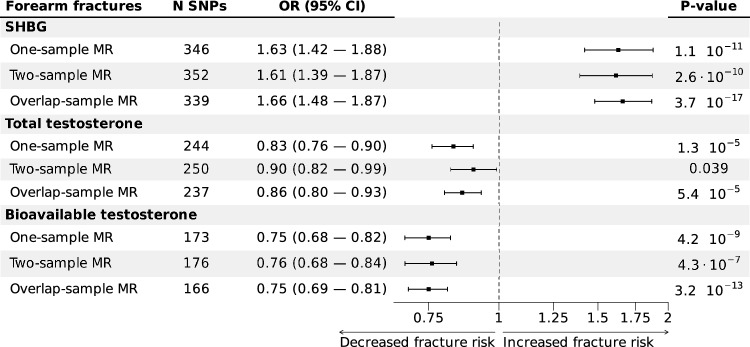
Forest plot of IVW MR associations of SHBG, TT, and BioT with forearm fractures. Inverse-variance weighted (IVW) causal effects of sex hormone-binding globulin (SHBG), total testosterone (TT), and bioavailable testosterone (BioT) on forearm fractures in women using three different outcome sets. One-sample MR consisting of data from the UK Biobank, two-sample MR consisting of data from Estonian Biobank (EstBB), The Trøndelag Health Study (HUNT), Umeå Fracture and Osteoporosis Study (UFO), and overlap-sample MR consisting of data from all four cohorts (UK Biobank, EstBB, HUNT, UFO). OR estimates and 95% CIs are given in OR change per 1 SD increase in SHBG, BioT, or TT. Abbreviations: IVW, inverse variance weighting; MR, Mendelian randomization; CI, confidence interval; SNPs, single-nucleotide polymorphisms

In the primary two-sample MR analyses, using an IVW method, we observed that high SHBG (OR per SD increase: 1.61, 95% confidence intervals (CIs) 1.39–1.87, *P* = 2.6 × 10^–10^) and low BioT (OR per SD increase: 0.76, 95% CI 0.68–0.84, *P* = 4.3 × 10^–07^) were causally associated with an increased risk of forearm fracture, while the inverse causal association of TT with fracture risk was comparatively weaker (OR per SD increase 0.90, 95% CI 0.82–0.99, 0.039) (Fig. 1, Supplementary Table S3). Analyses using UK Biobank in a one-sample MR setting and combined analysis of all four cohorts (overlap-sample MR) revealed similar effect estimates (Fig. 1, Supplementary Table S3).

When applying several alternative MR methodologies (Weighted median MR, Weighted mode MR, MR Egger, MR-PRESSO, MR LASSO, MR RAPS, and Steiger filtered analyses) in sensitivity analyses, the effect estimates for the causal associations with forearm fractures remained consistent (Supplementary Table S3). After excluding SNPs associated with potential confounders, the causal associations with forearm fractures also remained consistent (Supplementary Table S5). Scatter and funnel plots displayed moderate heterogeneity (Supplementary Fig. S3-S4), which also overall was confirmed by I2 index and the Q heterogeneity tests (Supplementary Table S3); however, the balanced distribution of individual estimates around the overall estimates suggests balanced pleiotropy, a finding supported by MR Egger analyses.

Analyses of three SHBG cis-SNPs yielded comparable and statistically significant Wald ratios in the two-sample and mixed-sample settings with slightly lower estimates in the one-sample setting. Combined analysis of all three cis-SNPs produced an SHBG IVW OR of 1.59 (95% CI 1.16–2.16) in the two-sample setting, closely matching estimates from analyses using all SHBG signals.

### Effect of TT, bioT, and SHBG on BMD

**Fig. 2:**
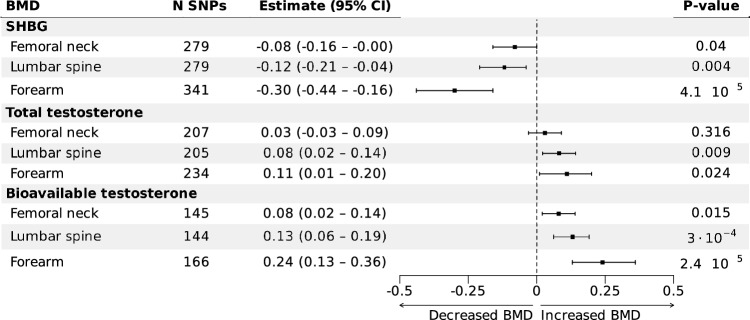
Forest plot of IVW MR associations of SHBG, TT, and BioT with bone mineral density. Inverse-variance weighted (IVW) causal effects of sex hormone-binding globulin (SHBG), total testosterone (TT), and bioavailable testosterone (BioT) on three different bone mineral density (BMD) measurements in women. Estimates and 95% CIs are given in SD change of BMD per 1 SD increase in SHBG, BioT, or TT. Abbreviations: SD, standard deviation; MR, Mendelian randomization; CI, confidence interval; SNPs, single-nucleotide polymorphisms

Analyses of BMD were in agreement with the effects seen on forearm fractures. In two-sample MR IVW analyses, BioT demonstrated a positive causal association with FN-BMD, LS-BMD, and FA-BMD, while SHBG exhibited an inverse causal relationship with all BMD measurements (Fig. 2, Supplementary Table S4). TT had somewhat smaller effect estimates and showed a significant positive causal association with FA-BMD and LS-BMD, but not with FN-BMD (Fig. 2, Supplementary Table S4). For all exposures, the effect estimates for FA-BMD were consistently larger than for LS-BMD and the effect estimates for LS-BMD were larger than for FN-BMD.

Sensitivity analyses employing multiple MR methodologies revealed a similar trend, albeit with some variations in estimated effect sizes (Supplementary Table S4). Upon excluding SNPs associated with potential confounders, the significant causal estimates observed in the primary analyses remained essentially stable (Supplementary Table S5). Visual examination of scatter and funnel plots for BMD outcomes revealed greater heterogeneity compared to forearm fracture analyses. Nevertheless, the overall balanced distribution of estimates implies balanced pleiotropy, a conclusion supported by MR Egger analyses (Supplementary Fig. S3-S4).

Analysis using the three SHBG cis-SNPs did not reveal statistically significant results for FN-BMD or LS-BMD in either individual or combined analyses. For FA-BMD analyses, one of the three SNPs (rs1799941) showed a statistically significant association (*P*-value: 0.014). Combined analyses of all three SNPs suggested a trend towards statistical significance, with a causal effect estimate of − 0.25 SD BMD change per 1 SD increase in SHBG (95% CI − 0.53 to 0.03, *P*-value: 0.074), which is comparable to estimates observed with trans-SNPs.

## Discussion

The role of androgens for bone health in females is unclear. We, herein, used female-specific genetic instruments to determine the effect of androgens and SHBG for forearm fracture risk specifically in females. We demonstrate that increased SHBG and decreased BioT and TT levels are causally linked with increased forearm fracture risk and lower BMD in women.

In the present study, we opted to examine forearm fractures as our fracture outcome based on a several considerations. First, among osteoporotic fractures, forearm fractures are notably more frequent and emerge earlier in women [68], making them the most suitable for achieving robust analytical power in our European cohorts, where participants’ average baseline age varied between 44 and 68 years. Secondly, both preclinical [5–7] and clinical data [18, 69] suggest that testosterone may preferentially affect trabecular bone. A majority of forearm fractures occurs in the distal forearm, which is a site of predominantly trabecular bone [70, 71], thus forearm fracture could likely be more susceptible to differences in androgen levels. Third, in a prior MR study, we established that decreased concentration of dehydroepiandrosterone sulfate (DHEAS), an adrenal sex steroid precursor, causes an increased risk of forearm fractures in females [72]. As there is an association of genetically predicted TT and BioT with genetically predicted DHEAS levels in women [41], we hypothesized that there is a likely upstream effect of DHEAS on TT, potentially influencing the risk of forearm fracture.

Though our study uniquely leverages both female-specific testosterone-related exposures and female-specific forearm fracture outcomes in a two-sample MR setting, our estimates of SHBG and BioT agree with earlier sex-combined MR studies [39, 40]. Sun et al. used sex-combined exposures for SHBG, derived from the UK Biobank, in two-sample MR analyses evaluating different sex-combined fracture outcomes using data from the FinnGen consortium [40]. In analogy with our results, high SHBG was associated with increased forearm fracture risk in that sex-combined study (OR = 1.23 per SD increase) but the effect estimate was more moderate compared with the effect estimate from the present female-specific MR analyses (OR = 1.61 per SD increase). Based on the combined findings from these two studies, we propose that the causal effect of high SHBG on forearm fracture risk may be stronger in women compared with men.

Our MR analyses reveal that BioT, TT, and SHBG causally affect BMD in females, exerting the greatest influence on FA-BMD and the smallest or no effect on FN-BMD, with LS-BMD estimates somewhere in between. These results indicate that at least part of the effect of BioT, TT, and SHBG on forearm fracture risk could be mediated by BMD. An earlier sex-combined two-sample MR study of SHBG, including only 11 genetic markers, observed a marked effect on forearm BMD, similar in size to our own finding for SHBG, however, this previous study found no significant effect on femoral neck or lumbar spine BMD, possibly related to using fewer genetic instruments and non-sex-specific exposure data [35]. Likewise, a later MR study noted significant effects of SHBG on female heel estimated BMD, that, similarly to the forearm, is a distal trabecular-rich bone site [36].

The present study has several strengths. We used exclusively female-specific datasets for both the selection of genetic instruments and for the exposures and outcomes in a solid two-sample MR framework. We obtained genetic instruments from the largest available female-specific GWAS of testosterone and SHBG to date, ensuring solid genetic instruments. Our MR analyses used a unique female-specific outcome dataset with well-defined forearm fracture cases that was well powered to detect also moderate effect sizes. We explored the MR assumptions by using multiple sensitivity analyses more robust to pleiotropy, used Steiger filtering to avoid problems of reverse causality, and excluded SNPs associated with potential confounders to ascertain that our effect estimates were consistent and reliable. By leveraging the protein nature of SHBG, unlike the sex steroid testosterone, we were also able to implement MR analyses using only cis-SNPs. This approach, which minimizes the risk of pleiotropic confounding [73], yielded consistent effect size estimates, further validating our results.

There are also some limitations of our study. First, our study focused solely on individuals of white ancestry, limiting the generalizability of our results to populations of other ancestries. Second, the large number of SNPs led to considerable heterogeneity in all our analyses. To ensure robust estimates, we employed a random-effects model as our primary method and supplemented it with several secondary methods based on different assumptions. All methods consistently indicated a strong association between SHBG, BioT, and forearm fracture risk, even when excluding SNPs associated with several potential confounders and when analyzing only SHBG cis-SNPs. Still, residual confounding or pleiotropy cannot be completely ruled out. Third, menopausal status of participants at the time of forearm fracture was not known for some of the included cohorts, preventing adjustment or stratification by menopausal status in the MR analyses. However, with the median age at forearm fracture ranging from 58 to 64 years, most fractures likely occurred after menopause. Moreover, because genetic variants used in Mendelian randomization remain consistent throughout life, the associated hormonal effects may have influenced skeletal features and forearm fracture susceptibility throughout life, including crucial periods such as puberty, the menopausal transition period, and after menopause. Thus, although forearm fractures mainly occur after menopause, it is challenging using standard MR methodology to pinpoint at which life stage testosterone and SHBG levels exert the most significant impact on forearm fracture risk. Fourth, a few of our outcome GWAS datasets lacked some exposure SNPs, a problem that persisted even after proxy SNPs was included. Consequently, some of these SNPs had to be left out of our MR analyses, especially in analyses of the LS-BMD and FN-BMD data which are limited to HapMap variants. Though the absence of some exposure SNPs in the outcome GWAS dataset may impair statistical power and potentially increase variability in sensitivity analyses, we are confident that we have included a reasonable number of SNPs to ensure the validity of our MR analyses.

In conclusion, our results establish a causal relationship of genetically predicted increases in SHBG and decreases in BioT with significantly increased forearm fracture risk in women. These effects of SHBG and BioT on forearm fracture risk were also associated with marked effects on forearm BMD, as well as more moderate effects on lumbar spine and femoral neck BMD in women. The impact of TT on these bone health outcomes appears to be less pronounced. Based on these results and previous studies, we propose that androgens and SHBG levels are important for women’s bone health of the distal forearm and that high SHBG and low BioT may serve as predictors for forearm fracture risk.

## Supplementary Information

Below is the link to the electronic supplementary material.

Supplementary file1 (PDF 4392 KB)

## Data Availability

Access to UK biobank data can be obtained by application to UK biobank (https://www.ukbiobank.ac.uk/). Access to individual-level data from HUNT requires collaboration with a Norwegian principal investigator. Researchers wishing to access HUNT data must apply through the HUNT Research Centre (https://www.ntnu.edu/hunt) and secure project approval from the Regional Committee for Medical and Health Research Ethics. Information on the application and conditions for data access in HUNT is available at https://www.ntnu.edu/hunt/data. Individual level Estonian Biobank data are available under restricted access administered by the Estonian Genome Centre of the University of Tartu (EGCUT) in accordance with the regulations of the Estonian Human Genes Research Act. Access can be obtained by application at https://genomics.ut.ee/en. For information on accessing data from the UFO, contact Ulrika Pettersson-Kymmer (ulrika.pettersson@umu.se). Summary statistics for bone mineral density measurements used in this article is publicly available via GEFOS: http://www.gefos.org.

## References

[1] Borgström F, Karlsson L, Ortsäter G, Norton N, Halbout P, Cooper C, Lorentzon M, McCloskey EV, Harvey NC, Javaid MK, Kanis JA, Cooper C, Reginster J-Y, Ferrari S, Halbout P, for the International Osteoporosis Foundation (2020) Fragility fractures in Europe: burden, management and opportunities. Arch Osteoporos 15:59. 10.1007/s11657-020-0706-y 32306163 10.1007/s11657-020-0706-y PMC7166207

[2] Budhia S, Mikyas Y, Tang M, Badamgarav E (2011) Osteoporotic fractures a systematic review of US healthcare costs and resource utilization. Pharmacoeconomics 30:147–170. 10.2165/11596880-000000000-00000 10.2165/11596880-000000000-00000 22187933

[3] Torgerson DJ, Bell-Syer SEM (2001) Hormone replacement therapy and prevention of nonvertebral fractures: a meta-analysis of randomized trials. JAMA 285:2891–2897. 10.1001/jama.285.22.2891 11401611 10.1001/jama.285.22.2891

[4] Manolagas SC, O’Brien CA, Almeida M (2013) The role of estrogen and androgen receptors in bone health and disease. Nat Rev Endocrinol 9:699–712. 10.1038/nrendo.2013.179 24042328 10.1038/nrendo.2013.179 PMC3971652

[5] Tivesten Å, Movérare-Skrtic S, Chagin A, Venken K, Salmon P, Vanderschueren D, Sävendahl L, Holmäng A, Ohlsson C (2004) Additive protective effects of estrogen and androgen treatment on trabecular bone in ovariectomized rats. J Bone Miner Res 19:1833–1839. 10.1359/JBMR.040819 15476584 10.1359/JBMR.040819

[6] Määttä JA, Büki KG, Ivaska KK, Nieminen-Pihala V, Elo TD, Kähkönen T, Poutanen M, Härkönen P, Väänänen K (2013) Inactivation of the androgen receptor in bone-forming cells leads to trabecular bone loss in adult female mice. Bonekey Rep 2:440. 10.1038/bonekey.2013.174 24422138 10.1038/bonekey.2013.174 PMC3844973

[7] Lin P-W, Lan K-C, Tsai M-Y, Shyr C-R, Chang C, Huang K-E, Kang H-Y (2018) The differential effects of sex hormones on the bone metabolism in mice with androgen receptor deficiency. Adapt Med 10:143–154. 10.4247/AM.2018.ABI217

[8] Bertelloni S, Meriggiola MC, Dati E, Balsamo A, Baroncelli GI (2017) Bone mineral density in women living with complete androgen insensitivity syndrome and intact testes or removed gonads. Sex Dev 11:182–189. 10.1159/000477599 28715798 10.1159/000477599

[9] Gava G, Mancini I, Orsili I, Bertelloni S, Alvisi S, Seracchioli R, Meriggiola MC (2019) Bone mineral density, body composition and metabolic profiles in adult women with complete androgen insensitivity syndrome and removed gonads using oral or transdermal estrogens. Eur J Endocrinol 181:711–718. 10.1530/EJE-19-0383 31491747 10.1530/EJE-19-0383

[10] Narinx N, David K, Walravens J, Vermeersch P, Claessens F, Fiers T, Lapauw B, Antonio L, Vanderschueren D (2022) Role of sex hormone-binding globulin in the free hormone hypothesis and the relevance of free testosterone in androgen physiology. Cell Mol Life Sci 79:543. 10.1007/s00018-022-04562-1 36205798 10.1007/s00018-022-04562-1 PMC11803068

[11] Slemenda C, Longcope C, Peacock M, Hui S, Johnston CC (1996) Sex steroids, bone mass, and bone loss. A prospective study of pre-, peri-, and postmenopausal women. J Clin Invest 97:14–21. 10.1172/JCI118382 8550826 10.1172/JCI118382 PMC507057

[12] Cummings SR, Browner WS, Bauer D, Stone K, Ensrud K, Jamal S, Ettinger B (1998) Endogenous hormones and the risk of hip and vertebral fractures among older women. N Engl J Med 339:733–738. 10.1056/NEJM199809103391104 9731089 10.1056/NEJM199809103391104

[13] Lee JS, LaCroix AZ, Wu L, Cauley JA, Jackson RD, Kooperberg C, Leboff MS, Robbins J, Lewis CE, Bauer DC, Cummings SR (2008) Associations of serum sex hormone-binding globulin and sex hormone concentrations with hip fracture risk in postmenopausal women. J Clin Endocrinol Metab 93:1796–1803. 10.1210/jc.2007-2358 18334588 10.1210/jc.2007-2358 PMC2386277

[14] Cauley JA, LaCroix AZ, Robbins JA, Larson J, Wallace R, Wactawski-Wende J, Chen Z, Bauer DC, Cummings SR, Jackson R (2009) Baseline serum estradiol and fracture reduction during treatment with hormone therapy: the women’s health initiative randomized trial. Osteoporos Int 21:167. 10.1007/s00198-009-0953-7 19436934 10.1007/s00198-009-0953-7 PMC2787820

[15] van Geel TACM, Geusens PP, Winkens B, Sels J-PJE, Dinant G-J (2009) Measures of bioavailable serum testosterone and estradiol and their relationships with muscle mass, muscle strength and bone mineral density in postmenopausal women: a cross-sectional study. Eur J Endocrinol 160:681–687. 10.1530/EJE-08-0702 19174532 10.1530/EJE-08-0702

[16] Finigan J, Gossiel F, Glüer CC, Felsenberg D, Reid DM, Roux C, Eastell R (2012) Endogenous estradiol and the risk of incident fracture in postmenopausal women: the OPUS study. Calcif Tissue Int 91:59–68. 10.1007/s00223-012-9611-8 22644322 10.1007/s00223-012-9611-8

[17] Greendale GA, Edelstein S, Barrett-Connor E (1997) Endogenous sex steroids and bone mineral density in older women and men: the Rancho Bernardo study. J Bone Miner Res 12:1833–1843. 10.1359/jbmr.1997.12.11.1833 9383688 10.1359/jbmr.1997.12.11.1833

[18] Khosla S, Melton LJ, Atkinson EJ, O’Fallon WM, Klee GG, Riggs BL (1998) Relationship of serum sex steroid levels and bone turnover markers with bone mineral density in men and women: a key role for bioavailable estrogen. J Clin Endocrinol Metab 83:2266–2274. 10.1210/jcem.83.7.4924 9661593 10.1210/jcem.83.7.4924

[19] Khosla S, Riggs BL, Robb RA, Camp JJ, Achenbach SJ, Oberg AL, Rouleau PA, Melton LJ III (2005) Relationship of volumetric bone density and structural parameters at different skeletal sites to sex steroid levels in women. J Clin Endocrinol Metab 90:5096–5103. 10.1210/jc.2005-0396 15998772 10.1210/jc.2005-0396 PMC1352154

[20] Cauley JA, Robbins J, Chen Z, Cummings SR, Jackson RD, LaCroix AZ, LeBoff M, Lewis CE, McGowan J, Neuner J, Pettinger M, Stefanick ML, Wactawski-Wende J, Watts NB, for the Women’s Health Initiative Investigators (2003) Effects of estrogen plus progestin on risk of fracture and bone mineral densitythe women’s health initiative randomized trial. JAMA 290:1729–1738. 10.1001/jama.290.13.1729 14519707 10.1001/jama.290.13.1729

[21] Rariy CM, Ratcliffe SJ, Weinstein R, Bhasin S, Blackman MR, Cauley JA, Robbins J, Zmuda JM, Harris TB, Cappola AR (2011) Higher serum free testosterone concentration in older women is associated with greater bone mineral density, lean body mass, and total fat mass: the cardiovascular health study. J Clin Endocrinol Metab 96:989–996. 10.1210/jc.2010-0926 21289255 10.1210/jc.2010-0926 PMC3070250

[22] Yee ML, Hau R, Taylor A, Guerra M, Guerra P, Darzins P, Gilfillan C (2020) Sarcopenia in women with hip fracture: a comparison of hormonal biomarkers and their relationship to skeletal muscle mass and function. Osteoporosis Sarcopenia 6:139–145. 10.1016/j.afos.2020.06.001 33102808 10.1016/j.afos.2020.06.001 PMC7573494

[23] Murphy S, Khaw KT, Sneyd MJ, Compston JE (1992) Endogenous sex hormones and bone mineral density among community-based postmenopausal women. Postgrad Med J 68:908–913. 10.1136/pgmj.68.805.908 1494513 10.1136/pgmj.68.805.908 PMC2399470

[24] Chapurlat RD, Garnero P, Bréart G, Meunier PJ, Delmas PD (2000) Serum estradiol and sex hormone-binding globulin and the risk of hip fracture in elderly women: the EPIDOS study. J Bone Miner Res 15:1835–1841. 10.1359/jbmr.2000.15.9.1835 10977003 10.1359/jbmr.2000.15.9.1835

[25] Sipilä S, Heikkinen E, Cheng S, Suominen H, Saari P, Kovanen V, Alén M, Rantanen T (2006) Endogenous hormones, muscle strength, and risk of fall-related fractures in older women. J Gerontol A Biol Sci Med Sci 61:92–96. 10.1093/gerona/61.1.92 16456199 10.1093/gerona/61.1.92

[26] Cauley JA, Danielson ME, Jammy GR, Bauer DC, Jackson R, Wactawski-Wende J, Chlebowski RT, Ensrud KE, Boudreau R (2017) Sex steroid hormones and fracture in a multiethnic cohort of women: the Women’s Health Initiative study (WHI). J Clin Endocrinol Metab 102:1538–1547. 10.1210/jc.2016-3589 28324031 10.1210/jc.2016-3589 PMC5443326

[27] Cauley JA, Ruppert K, Lian Y, Finkelstein JS, Karvonen-Gutierrez CA, Harlow SD, Lo JC, Burnett-Bowie S-AM, Karlamangla A, Greendale GA (2019) Serum sex hormones and the risk of fracture across the menopausal transition: study of women’s health across the nation. J Clin Endocrinol Metab 104:2412–2418. 10.1210/jc.2018-02047 30690517 10.1210/jc.2018-02047 PMC6505454

[28] Douchi T, Oki T, Yamasaki H, Kuwahata R, Nakae M, Nagata Y (2001) Relationship of androgens to muscle size and bone mineral density in women with polycystic ovary syndrome. Obstet Gynecol 98:445–449. 10.1016/S0029-7844(01)01450-8 11530127 10.1016/s0029-7844(01)01450-8

[29] Goderie-Plomp HW, van der Klift M, de Ronde W, Hofman A, de Jong FH, Pols HAP (2004) Endogenous sex hormones, sex hormone-binding globulin, and the risk of incident vertebral fractures in elderly men and women: the Rotterdam study. J Clin Endocrinol Metab 89:3261–3269. 10.1210/jc.2002-022041 15240601 10.1210/jc.2002-022041

[30] Liu S, Tian L, Xu P, Zhuang G, Zheng F, Tian J, Ning Q-L, Zhu B-F, Lu S-M, Yan H (2011) Analysis of correlation between blood biochemical indicators and bone mineral density of post-menopausal women. Mol Biol Rep 38:939–948. 10.1007/s11033-010-0187-y 20490690 10.1007/s11033-010-0187-y

[31] Fighera TM, Ziegelmann PK, Rasia da Silva T, Spritzer PM (2019) Bone mass effects of cross-sex hormone therapy in transgender people: updated systematic review and meta-analysis. J Endocr Soc 3:943–964. 10.1210/js.2018-00413 31020058 10.1210/js.2018-00413 PMC6469959

[32] Zhang H, Ma K, Li R-M, Li J-N, Gao S-F, Ma L-N (2022) Association between testosterone levels and bone mineral density in females aged 40–60 years from NHANES 2011–2016. Sci Rep 12:16426. 10.1038/s41598-022-21008-7 36180560 10.1038/s41598-022-21008-7 PMC9525583

[33] Lawlor DA, Harbord RM, Sterne JAC, Timpson N, Davey Smith G (2008) Mendelian randomization: using genes as instruments for making causal inferences in epidemiology. Stat Med 27:1133–1163. 10.1002/sim.3034 17886233 10.1002/sim.3034

[34] Mohammadi-Shemirani P, Chong M, Pigeyre M, Morton RW, Gerstein HC, Paré G (2020) Effects of lifelong testosterone exposure on health and disease using Mendelian randomization. Elife. 10.7554/eLife.58914 33063668 10.7554/eLife.58914 PMC7591257

[35] Qu Z, Jiang J, Yang F, Huang J, Zhao J, Yan S (2021) Genetically predicted sex hormone-binding globulin and bone mineral density: a Mendelian randomization study. Calcif Tissue Int 108:281–287. 10.1007/s00223-020-00770-8 33068140 10.1007/s00223-020-00770-8

[36] Qu Y, Xiao C, Wu X, Zhu J, Qin C, He L, Cui H, Zhang L, Zhang W, Yang C, Yao Y, Li J, Liu Z, Zhang B, Wang W, Jiang X (2023) Genetic correlation, shared loci, and causal association between sex hormone-binding globulin and bone mineral density: insights from a large-scale genomewide cross-trait analysis. J Bone Miner Res 38:1635–1644. 10.1002/jbmr.4904 37615194 10.1002/jbmr.4904

[37] Huang W, Xiao Y, Zhang L, Liu H (2024) The association between SHBG and osteoporosis: A NHANES cross-sectional study and a bidirectional Mendelian randomization. Calcif Tissue Int 114:237–245. 10.1007/s00223-023-01166-0 38051322 10.1007/s00223-023-01166-0

[38] Sun K, Li M, Wu Y, Wu Y, Zeng Y, Zhou S, Peng L, Shen B (2024) Exploring causal relationships between leukocyte telomere length, sex hormone-binding globulin levels, and osteoporosis using univariable and multivariable Mendelian randomization. Orthop Surg 16:320–328. 10.1111/os.13947 38084376 10.1111/os.13947 PMC10834216

[39] Yuan S, Wang L, Sun J, Yu L, Zhou X, Yang J, Zhu Y, Gill D, Burgess S, Denny JC, Larsson SC, Theodoratou E, Li X (2022) Genetically predicted sex hormone levels and health outcomes: phenome-wide Mendelian randomization investigation. Int J Epidemiol 51:1931–1942. 10.1093/ije/dyac036 35218343 10.1093/ije/dyac036 PMC9749729

[40] Sun K, Ming Y, Xu J, Wu Y, Zeng Y, Wu L, Li M, Shen B (2023) Assessing the casual association between sex hormone levels and fracture risk: a two-sample Mendelian randomization study. Orthop Surg 15:3065–3074. 10.1111/os.13881 37771125 10.1111/os.13881 PMC10694015

[41] Ruth KS et al (2020) Using human genetics to understand the disease impacts of testosterone in men and women. Nat Med 26:252–258. 10.1038/s41591-020-0751-5 32042192 10.1038/s41591-020-0751-5 PMC7025895

[42] Nethander M, Movérare-Skrtic S, Kämpe A, Coward E, Reimann E, Grahnemo L, Borbély É, Helyes Z, Funck-Brentano T, Cohen-Solal M, Tuukkanen J, Koskela A, Wu J, Li L, Lu T, Gabrielsen ME, Mägi R, Hoff M, Lerner UH, Henning P, Ullum H, Erikstrup C, Brunak S, Langhammer A, Tuomi T, Oddsson A, Stefansson K, Pettersson-Kymmer U, Ostrowski SR, Pedersen OBV, Styrkarsdottir U, Mäkitie O, Hveem K, Richards JB, Ohlsson C (2023) An atlas of genetic determinants of forearm fracture. Nat Genet 55:1820–1830. 10.1038/s41588-023-01527-3 37919453 10.1038/s41588-023-01527-3 PMC10632131

[43] Gill D, Georgakis MK, Walker VM, Schmidt AF, Gkatzionis A, Freitag DF, Finan C, Hingorani AD, Howson JMM, Burgess S, Swerdlow DI, Davey Smith G, Holmes MV, Dichgans M, Scott RA, Zheng J, Psaty BM, Davies NM (2021) Mendelian randomization for studying the effects of perturbing drug targets. Wellcome Open Res. 10.12688/wellcomeopenres.16544.2 33644404 10.12688/wellcomeopenres.16544.1 PMC7903200

[44] Estrada K et al (2012) Genome-wide meta-analysis identifies 56 bone mineral density loci and reveals 14 loci associated with risk of fracture. Nat Genet 44:491–501. 10.1038/ng.2249 22504420 10.1038/ng.2249 PMC3338864

[45] Zheng H-F et al (2015) Whole-genome sequencing identifies EN1 as a determinant of bone density and fracture. Nature 526:112–117. 10.1038/nature14878 26367794 10.1038/nature14878 PMC4755714

[46] Skrivankova VW, Richmond RC, Woolf BAR, Davies NM, Swanson SA, VanderWeele TJ, Timpson NJ, Higgins JPT, Dimou N, Langenberg C, Loder EW, Golub RM, Egger M, Smith GD, Richards JB (2021) Strengthening the reporting of observational studies in epidemiology using mendelian randomisation (STROBE-MR): explanation and elaboration. BMJ 375:n2233. 10.1136/bmj.n2233 34702754 10.1136/bmj.n2233 PMC8546498

[47] Sudlow C, Gallacher J, Allen N, Beral V, Burton P, Danesh J, Downey P, Elliott P, Green J, Landray M, Liu B, Matthews P, Ong G, Pell J, Silman A, Young A, Sprosen T, Peakman T, Collins R (2015) UK biobank: an open access resource for identifying the causes of a wide range of complex diseases of middle and old age. PLoS Med 12:e1001779. 10.1371/journal.pmed.1001779 25826379 10.1371/journal.pmed.1001779 PMC4380465

[48] UK Biobank (2019) UK Biobank Biomarker Project - companion document to accompany serum biomarker data. Version 1.0

[49] Rietveld CA et al (2013) GWAS of 126,559 individuals identifies genetic variants associated with educational attainment. Science 340:1467–1471. 10.1126/science.1235488 23722424 10.1126/science.1235488 PMC3751588

[50] Zhou S, Sosina OA, Bovijn J, Laurent L, Sharma V, Akbari P, Forgetta V, Jiang L, Kosmicki JA, Banerjee N, Morris JA, Oerton E, Jones M, LeBlanc MG, Idone V, Overton JD, Reid JG, Cantor M, Abecasis GR, Goltzman D, Greenwood CMT, Langenberg C, Baras A, Economides AN, Ferreira MAR, Hatsell S, Ohlsson C, Richards JB, Lotta LA (2023) Converging evidence from exome sequencing and common variants implicates target genes for osteoporosis. Nat Genet 55:1277–1287. 10.1038/s41588-023-01444-5 37558884 10.1038/s41588-023-01444-5

[51] Melzer D, Perry JRB, Hernandez D, Corsi A-M, Stevens K, Rafferty I, Lauretani F, Murray A, Gibbs JR, Paolisso G, Rafiq S, Simon-Sanchez J, Lango H, Scholz S, Weedon MN, Arepalli S, Rice N, Washecka N, Hurst A, Britton A, Henley W, van de Leemput J, Li R, Newman AB, Tranah G, Harris T, Panicker V, Dayan C, Bennett A, McCarthy MI, Ruokonen A, Jarvelin M-R, Guralnik J, Bandinelli S, Frayling TM, Singleton A, Ferrucci L (2008) A genome-wide association study identifies protein quantitative trait loci (pQTLs). PLoS Genet 4:e1000072. 10.1371/journal.pgen.1000072 18464913 10.1371/journal.pgen.1000072 PMC2362067

[52] Bowden J, Del Greco MF, Minelli C, Davey Smith G, Sheehan N, Thompson J (2017) A framework for the investigation of pleiotropy in two-sample summary data Mendelian randomization. Stat Med 36:1783–1802. 10.1002/sim.7221 28114746 10.1002/sim.7221 PMC5434863

[53] Bowden J, Davey Smith G, Haycock PC, Burgess S (2016) Consistent estimation in mendelian randomization with some invalid instruments using a weighted median estimator. Genet Epidemiol 40:304–314. 10.1002/gepi.21965 27061298 10.1002/gepi.21965 PMC4849733

[54] Hartwig FP, Davey Smith G, Bowden J (2017) Robust inference in summary data Mendelian randomization via the zero modal pleiotropy assumption. Int J Epidemiol 46:1985–1998. 10.1093/ije/dyx102 29040600 10.1093/ije/dyx102 PMC5837715

[55] Bowden J, Davey Smith G, Burgess S (2015) Mendelian randomization with invalid instruments: effect estimation and bias detection through Egger regression. Int J Epidemiol 44:512–525. 10.1093/ije/dyv080 26050253 10.1093/ije/dyv080 PMC4469799

[56] Verbanck M, Chen C-Y, Neale B, Do R (2018) Detection of widespread horizontal pleiotropy in causal relationships inferred from Mendelian randomization between complex traits and diseases. Nat Genet 50:693–698. 10.1038/s41588-018-0099-7 29686387 10.1038/s41588-018-0099-7 PMC6083837

[57] Rees JMB, Wood AM, Dudbridge F, Burgess S (2019) Robust methods in Mendelian randomization via penalization of heterogeneous causal estimates. PLoS ONE 14:e0222362. 10.1371/journal.pone.0222362 31545794 10.1371/journal.pone.0222362 PMC6756542

[58] Hemani G, Tilling K, Davey Smith G (2017) Orienting the causal relationship between imprecisely measured traits using GWAS summary data. PLoS Genet 13:e1007081. 10.1371/journal.pgen.1007081 29149188 10.1371/journal.pgen.1007081 PMC5711033

[59] Hemani G, Bowden J, Davey Smith G (2018) Evaluating the potential role of pleiotropy in Mendelian randomization studies. Hum Mol Genet 27:R195–R208. 10.1093/hmg/ddy163 29771313 10.1093/hmg/ddy163 PMC6061876

[60] Vandenput L et al (2022) Update of the fracture risk prediction tool FRAX: a systematic review of potential cohorts and analysis plan. Osteoporos Int 33:2103–2136. 10.1007/s00198-022-06435-6 35639106 10.1007/s00198-022-06435-6

[61] Elsworth B, Lyon M, Alexander T, Liu Y, Matthews P, Hallett J, Bates P, Palmer T, Haberland V, Smith GD, Zheng J, Haycock P, Gaunt TR, Hemani G (2020) The MRC IEU OpenGWAS data infrastructure. 2020.08.10.244293

[62] Burgess S, Bowden J, Fall T, Ingelsson E, Thompson SG (2017) Sensitivity analyses for robust causal inference from Mendelian randomization analyses with multiple genetic variants. Epidemiology 28:30–42. 10.1097/EDE.0000000000000559 27749700 10.1097/EDE.0000000000000559 PMC5133381

[63] Burgess S, Zuber V, Valdes-Marquez E, Sun BB, Hopewell JC (2017) Mendelian randomization with fine-mapped genetic data: choosing from large numbers of correlated instrumental variables. Genet Epidemiol 41:714–725. 10.1002/gepi.22077 28944551 10.1002/gepi.22077 PMC5725678

[64] Hemani G, Zheng J, Elsworth B, Wade KH, Haberland V, Baird D, Laurin C, Burgess S, Bowden J, Langdon R, Tan VY, Yarmolinsky J, Shihab HA, Timpson NJ, Evans DM, Relton C, Martin RM, Davey Smith G, Gaunt TR, Haycock PC (2018) The MR-Base platform supports systematic causal inference across the human phenome. Elife 7:e34408. 10.7554/eLife.34408 29846171 10.7554/eLife.34408 PMC5976434

[65] Yavorska OO, Burgess S (2017) MendelianRandomization: an R package for performing Mendelian randomization analyses using summarized data. Int J Epidemiol 46:1734–1739. 10.1093/ije/dyx034 28398548 10.1093/ije/dyx034 PMC5510723

[66] Brion M-JA, Shakhbazov K, Visscher PM (2013) Calculating statistical power in Mendelian randomization studies. Int J Epidemiol 42:1497–1501. 10.1093/ije/dyt179 24159078 10.1093/ije/dyt179 PMC3807619

[67] Shim H, Chasman DI, Smith JD, Mora S, Ridker PM, Nickerson DA, Krauss RM, Stephens M (2015) A multivariate genome-wide association analysis of 10 LDL subfractions, and their response to statin treatment, in 1868 Caucasians. PLoS ONE 10:e0120758. 10.1371/journal.pone.0120758 25898129 10.1371/journal.pone.0120758 PMC4405269

[68] Harvey N, Dennison E, Cooper C (2010) Osteoporosis: impact on health and economics. Nat Rev Rheumatol 6:99–105. 10.1038/nrrheum.2009.260 20125177 10.1038/nrrheum.2009.260

[69] Bretherton I, Ghasem-Zadeh A, Leemaqz SY, Seeman E, Wang X, McFarlane T, Spanos C, Grossmann M, Zajac JD, Cheung AS (2022) Bone microarchitecture in transgender adults: a cross-sectional study. J Bone Miner Res 37:643–648. 10.1002/jbmr.4497 34981566 10.1002/jbmr.4497 PMC9305455

[70] Link TM, Bauer J, Kollstedt A, Stumpf I, Hudelmaier M, Settles M, Majumdar S, Lochmüller E-M, Eckstein F (2004) Trabecular bone structure of the distal radius, the calcaneus, and the spine: which site predicts fracture status of the spine best? Invest Radiol 39:487–497. 10.1097/01.rli.0000129154.50654.4e 15257210 10.1097/01.rli.0000129154.50654.4e

[71] Nethander M, Pettersson-Kymmer U, Vandenput L, Lorentzon M, Karlsson M, Mellström D, Ohlsson C (2020) BMD-related genetic risk scores predict site-specific fractures as well as trabecular and cortical bone microstructure. J Clin Endocrinol Metab 105:e1344–e1357. 10.1210/clinem/dgaa082 32067027 10.1210/clinem/dgaa082 PMC7069346

[72] Quester J, Nethander M, Eriksson A, Ohlsson C (2022) Endogenous DHEAS is causally linked with lumbar spine bone mineral density and forearm fractures in women. J Clin Endocrinol Metab 107:e2080–e2086. 10.1210/clinem/dgab915 34935937 10.1210/clinem/dgab915 PMC9016453

[73] Patel A, Gill D, Newcombe P, Burgess S (2023) Conditional inference in cis-mendelian randomization using weak genetic factors. Biometrics 79:3458–3471. 10.1111/biom.13888 37337418 10.1111/biom.13888 PMC7615409

