# Supplementary material for: High SHBG and Low Bioavailable Testosterone are Strongly Causally Associated with Increased Forearm Fracture Risk in Women: An MR Study Leveraging Novel Female-Specific Data
Source: Calcif Tissue Int. 2024 Oct 16;115(5):648–60. doi: 10.1007/s00223-024-01301-5 (PMC11531422; doi:10.1007/s00223-024-01301-5)
Supplement: Supplementary file 1 — Supplementary file1 (PDF 4392 KB) [file 223_2024_1301_MOESM1_ESM.pdf]

Supplementary to "High SHBG and Low Bioavailable Testosterone Are Strongly Causally Associated with Increased Forearm Fracture Risk in Women: An MR Study Leveraging Novel Female-Specific Data"

Johan Quester, Maria Nethander, Eivind Coward, Ene Reimann, Reedik Mägi, Ulrika Pettersson-Kymmer, Kristian Hveem, Claes Ohlsson

Name	Content	Page
Supplementary Table S1	Descriptive study design of individual forearm fracture studies	2
Supplementary Table S2	Descriptive baseline statistics for women in individual forearm fracture cohorts	3
Supplementary Table S3	Mendelian randomization results of the causal association between bioavailable testosterone, SHBG or total testosterone and forearm fractures in women.	4
Supplementary Table S4	Two sample Mendelian randomization results of the causal association between bioavailable testosterone, sex hormone binding globuline (SHBG) or total testosterone and BMD.	5
Supplementary Tables S5	Mendelian randomization results of (A) Forearm fractures and (B) BMD after removing genetic variants associated with potential confounders** at a P-value $< 7 \times 10^{-5}$	6
Supplementary Table S6	Details of GWAS used to identify genetic variants associated with potential confounders for forearm fractures and bone mineral density.	7
Supplementary Table S7	Results from Mendelian randomization analyses of three individual SHBG cis-SNPs (Wald ratios) and combined analyses with all three SHBG cis-SNPs (IVW MR)	8
Supplementary Figure S1	Flow chart of study methodology.	9
Supplementary Figure S2	Power curves of MR analyses of forearm fractures	10
Supplementary Figure S3	MR Scatter plots	11
Supplementary Figure S4	MR Funnel plots	12

Supplementary Table S1: Descriptive study design of individual forearm fracture studies

Short name	UKBB	UFO	HUNT	EstBB
Full name	UK Biobank	Umeå Fracture and Osteoporosis Study	The Trøndelag Health Study	Estonian Biobank
Area of Origin	UK	Sweden/Umeå	Norway/Trøndelag	Estonia/Tartu
Ethnicity	North-Western European	Northern European	Northern European	Northern European
Study Type	Population based	Population based	Population based	Population based
Number of women with phenotype information	237,572	1,706	36,028	73,617
Study Design	Cohort study. Both prevalent and incident forearm fractures were identified by ICD codes (ICD10, S52; ICD9, 813) from registers, including cases over 30 years old. Controls were individuals from the same cohort without a history of forearm fracture. Height and weight were derived from the UK Biobank baseline visit. For fracture cases, age was the time of the first forearm fracture; for controls, it was the age of censoring (death, emigration or time of evaluation). Data are pseudonymized and participants cannot be identified by the researcher accessing the data. Current fracture data were censored as of 30.09.2021, and analyzed by author Maria Nethander using secure, limited-access servers. The UK Biobank has ethical approval from the Northwest Multicentre Research Ethics Committee (11/NW/0382). Informed written consent was obtained from all participants. The present research was approved by the UK Biobank Research and Access Committee (application number 51784).	Nested-Case-Control study. Both prevalent and incident forearm fractures were identified by ICD codes (ICD10, S52; ICD9, 813). Fracture cases >30 years were included. All fracture cases were also confirmed by radiological report and or medical records. The fracture data employed in the current analysis were collected up until 31.12.2008. All data are pseudonymized and biobank participants cannot be identified by the researcher accessing the data. The data were analysed by author Maria Nethander using secured limitided access servers. The UFO study was approved by the local research ethics committee at Umeå University (Umu dnr 03-426; EPN 2012-254-32M; 2011/32-32M; 2011-251-32M). Informed written consent was obtained from all participants.	Cohort study. Both prevalent and incident forearm fractures were identified by ICD codes (ICD10, S52; ICD9, 813) from registers. Fracture cases >30 years were included. Controls were defined as individuals from the same cohorts, without a history of forearm fracture. For fracture cases, age was the time for first forearm fracture and for controls the age was the age at the latest available HUNT visit. The current fracture data were censored as of 30.11.2020, and analysed by author Eivind Coward using secured limited access servers. The researcher has only access to pseudonymized data and cannot identify biobank participants. Participation in the HUNT Study is based on informed written consent and the study has been approved by the Regional Ethics Committee for Medical Research in Norway (REK 2015/615; REK 2014/144).	Cohort study. Both prevalent and incident forearm fractures were identified by ICD codes (ICD10, S52; ICD9, 813) from registers. Fracture cases >30 years were included. Controls were defined as individuals from the same cohorts, without a history of forearm fracture. Height and weight were derived from the latest visit. For fracture cases, age was the time for first forearm fracture and for controls the age was age at recruitment to EstBB. The currently used data were accessed 19.05.2020 by author Ene Reimann via secured servers. The researcher has only access to pseudonymized data and cannot identify biobank participants. All data analyses were conducted in secured limited access servers (HPC University of Tartu). Estonian Biobank has ethical approval from the Estonian Commitee on Bioethics and Human Research at the Ministry of Social Affairs (No 1.1-12/624). Written informed consent for participation was obtained from all study subjects.
Study Description	Between 2006 and 2010, the UK Biobank recruited over 500,000 participants aged 40–69 from various regions across the United Kingdom. Participants contributed extensive health and lifestyle information through digital questionnaires and physical exams, and provided blood, urine, and saliva samples for future analysis. They also consented to longitudinal health monitoring through linkage to health-related records. The UK Biobank genetic data includes genotypes for a total of 488,377 participants.	The Umeå Fracture and Osteoporosis (UFO) study is a nested case-control study investigating associations between genes, lifestyle and osteoporotic fractures. This cohort is sampled from the prospective and population based Northern Sweden Health and Disease Study (NSDHS) cohort, initiated to assess risk factors for diabetes and cardiovascular disease, that consists of blood samples and lifestyle data from around 100,000 individuals. The UFO-forearm fracture study recruited participants in 2008. Forearm fracture cases were identified by merging the NSHDS cohort with medical records and radiographic reports. Each forearm fracture case is compared with 1 control selected from the NSHDS cohort and matched for gender and age.	HUNT is a population-based health study in Trøndelag County, Norway that comprises data and samples obtained through four population studies between 1984 and 2019. The study acquired health-related information via questionnaires, interviews, and clinical assessments. Participants also consented to longitudinal linkage to health and administrative registries in Norway and to information from medical records. HUNT has enrolled over 229,000 adults aged 20 or older, with 95,000 providing at least one biological specimen and 88,000 contributing DNA samples for genetic research. Genome-wide genotyping has been completed for approximately 70,000 individuals.	The Estonian Biobank cohort is a volunteer-based sample of the Estonian resident adult population (aged ≥18 years) recruited between 2002 and 2020. Estonians represent 83%, Russians 14%, and other nationalities 3% of all participants. The current number of participants is close to 200,000 and represents a large proportion, > 15 %, of the Estonian adult population, making it ideally suited to population-based studies. General practitioners (GPs) and medical personnel in the special recruitment offices have recruited participants throughout the country. At baseline, the GPs performed a standardized health examination of the participants, who also donated blood samples for DNA, white blood cells and plasma tests and filled out a 16-module questionnaire on health-related topics such as lifestyle, diet and clinical diagnoses described in WHO ICD-10. A significant part of the cohort has whole genome sequencing (3000), whole exome sequencing (2500), genome-wide single nucleotide polymorphism (SNP) array data (>200 000) and/or NMR metabolome data (11 000) available. The data are continuously updated through periodical linking to national electronic databases and registries. A part of the cohort has been re-contacted for follow-up purposes and resampling, and targeted invitations are possible for specific purposes, for example people with a specific diagnosis. For the current study data freeze including approximately 150,000 gene donors was applied, which resulted in including 109410 gene donors into the study.
References	PMID: 25826379	PMID32067027, PMID20464545	PMID36777998	PMID24518929; PMID24518929

Supplementary Table S2: Descriptive baseline statistics for women in individual forearm fracture cohorts

Short name	Trait	N	mean	SD
UKBB	Age (yrs)	237,572	67.7	8.2
	Height (cm)		162.6	6.2
	Weight (kg)		71.4	13.9
UFO	Age (yrs)	1,706	61.5	6.7
	Height (cm)		163.7	5.8
	Weight (kg)		67.9	10.9
HUNT	Age (yrs)	36,028	52.7	17.5
	Height (cm)		164.1	6.5
	Weight (kg)		72.5	13.7
EstBB	Age (yrs)	73,617	43.6	15.9
	Height (cm)		166.0	6.4
	Weight (kg)		70.9	15.0

Supplementary Table S3: Mendelian randomization results of the causal association between bioavailable testosterone, SHBG or total testosterone and forearm fractures in women.

**MR analysis type**	**One-sample MR**			**Two-sample MR**			**Mixed-sample MR**		
**Cohort**	UK Biobank			EstBB, HUNT, UFO			EstBB, HUNT, UFO, UK Biobank		
**Fracture**	Forearm			Forearm			Forearm		
**N total / cases**	237,572 / 11,564			111,351 / 8,823			348,923 / 20,387		
**Exposure**	bioT	SHBG	TT	bioT	SHBG	TT	bioT	SHBG	TT
**Primary MR analyses**									
**N variants**	173	346	244	176	352	250	166	339	237
**Inverse variance weighted MR**									
OR (95% CI)	0.75 (0.68 – 0.82)	1.63 (1.42 – 1.88)	0.83 (0.76 – 0.90)	0.76 (0.68 – 0.84)	1.61 (1.39 – 1.87)	0.90 (0.82 – 0.99)	0.75 (0.69 – 0.81)	1.66 (1.48 – 1.87)	0.86 (0.80 – 0.93)
P	**4.2e-09**	**1.1e-11**	**1.3e-05**	**4.3e-07**	**2.6e-10**	**0.039**	**3.2e-13**	**3.7e-17**	**5.4e-05**
Q	238	434	345	210	398	335	223	495	402
Q P	**6.50E-04**	**7.70E-04**	**1.80E-05**	**0.035**	**0.044**	**2.20E-04**	**0.002**	**4.80E-08**	**8.50E-11**
I^2	27.7	20.5	29.6	16.8	11.7	25.7	26	31.8	41.3
**Weighted median MR**									
OR (95% CI)	0.79 (0.66 – 0.94)	1.33 (1.02 – 1.73)	0.81 (0.72 – 0.92)	0.74 (0.62 – 0.88)	1.52 (1.20 – 1.91)	0.87 (0.73 – 1.04)	0.76 (0.66 – 0.87)	1.42 (1.15 – 1.76)	0.85 (0.77 – 0.93)
P	**0.008**	**0.035**	**8.3e-04**	**5.4e-04**	**4.1e-04**	0.124	**1.4e-04**	**9.9e-04**	**7.1e-04**
**Weighted mode MR**									
OR (95% CI)	0.83 (0.66 – 1.03)	1.29 (1.04 – 1.61)	0.68 (0.59 – 0.79)	0.73 (0.61 – 0.87)	1.51 (1.21 – 1.87)	0.91 (0.75 – 1.09)	0.76 (0.65 – 0.89)	1.39 (1.16 – 1.66)	0.76 (0.68 – 0.85)
P	0.087	**0.022**	**1.5e-06**	**5.9e-04**	**2.7e-04**	0.288	**6.4e-04**	**3.7e-04**	**4.5e-06**
**MR Egger**									
OR (95% CI)	0.68 (0.56 – 0.83)	1.41 (1.11 – 1.79)	0.71 (0.61 – 0.83)	0.72 (0.60 – 0.87)	1.47 (1.18 – 1.83)	0.94 (0.78 – 1.12)	0.70 (0.60 – 0.81)	1.48 (1.21 – 1.80)	0.80 (0.70 – 0.91)
P	**2.0e-04**	**0.005**	**2.3e-05**	**9.3e-04**	**6.3e-04**	0.472	**5.2e-06**	**1.3e-04**	**0.001**
Intercept P	0.325	0.142	**0.020**	0.545	0.256	0.633	0.273	0.135	0.174
**MR PRESSO**									
OR (95% CI)	0.79 (0.72 – 0.87)	1.63 (1.42 – 1.88)	0.83 (0.76 – 0.90)	0.76 (0.68 – 0.84)	1.61 (1.39 – 1.87)	0.91 (0.82 – 1.00)	0.75 (0.70 – 0.81)	1.66 (1.48 – 1.87)	0.85 (0.80 – 0.91)
P	**2.2e-06**	**1.1e-11**	**1.3e-05**	**4.3e-07**	**2.6e-10**	0.051	**9.1e-12**	**1.1e-15**	**2.9e-06**
N var. excl.	3	0	0	0	0	1	1	0	5
**MR LASSO**									
OR (95% CI)	0.79 (0.72 – 0.86)	1.52 (1.34 – 1.73)	0.86 (0.80 – 0.93)	0.74 (0.67 – 0.82)	1.60 (1.39 – 1.84)	0.90 (0.83 – 0.98)	0.77 (0.72 – 0.83)	1.60 (1.45 – 1.77)	0.89 (0.83 – 0.94)
P	**6.5e-08**	**1.4e-10**	**1.3e-04**	**4.0e-09**	**5.8e-11**	**0.021**	**3.5e-13**	**5.3e-20**	**9.6e-05**
N var. excl.	11	13	14	8	9	14	10	32	17
**MR RAPS**									
OR (95% CI)	0.76 (0.69 – 0.84)	1.61 (1.39 – 1.87)	0.84 (0.77 – 0.91)	0.75 (0.67 – 0.84)	1.62 (1.39 – 1.89)	0.91 (0.82 – 1.00)	0.76 (0.70 – 0.82)	1.67 (1.47 – 1.90)	0.86 (0.80 – 0.92)
P	**7.3e-08**	**2.6e-10**	**3.1e-05**	**3.4e-07**	**3.7e-10**	0.056	**6.8e-12**	**1.1e-15**	**2.5e-05**
**Steiger filtered analyses**									
**N variants**	173	346	244	176	351	250	166	339	237
**Inverse variance weighted MR**									
OR (95% CI)	0.75 (0.68 – 0.82)	1.63 (1.42 – 1.88)	0.83 (0.76 – 0.90)	0.76 (0.68 – 0.84)	1.61 (1.39 – 1.86)	0.90 (0.82 – 0.99)	0.75 (0.69 – 0.81)	1.66 (1.48 – 1.87)	0.86 (0.80 – 0.93)
P	**4.2e-09**	**1.1e-11**	**1.3e-05**	**4.3e-07**	**2.6e-10**	**0.039**	**3.2e-13**	**3.7e-17**	**5.4e-05**
**Weighted median MR**									
OR (95% CI)	0.79 (0.66 – 0.94)	1.33 (1.03 – 1.72)	0.81 (0.72 – 0.91)	0.74 (0.62 – 0.87)	1.52 (1.21 – 1.90)	0.87 (0.74 – 1.03)	0.76 (0.66 – 0.87)	1.42 (1.16 – 1.75)	0.85 (0.77 – 0.93)
P	**0.008**	**0.029**	**4.5e-04**	**4.1e-04**	**3.0e-04**	0.118	**1.2e-04**	**7.5e-04**	**7.4e-04**
**Weighted mode MR**									
OR (95% CI)	0.83 (0.67 – 1.02)	1.29 (1.04 – 1.61)	0.68 (0.58 – 0.79)	0.73 (0.61 – 0.88)	1.51 (1.21 – 1.87)	0.91 (0.75 – 1.09)	0.76 (0.65 – 0.89)	1.39 (1.17 – 1.65)	0.76 (0.67 – 0.85)
P	0.071	**0.023**	**1.7e-06**	**0.001**	**2.7e-04**	0.293	**9.4e-04**	**2.0e-04**	**9.4e-06**
**MR Egger**									
OR (95% CI)	0.68 (0.56 – 0.83)	1.41 (1.11 – 1.79)	0.71 (0.61 – 0.83)	0.72 (0.60 – 0.87)	1.48 (1.19 – 1.84)	0.94 (0.78 – 1.12)	0.70 (0.60 – 0.81)	1.48 (1.21 – 1.80)	0.80 (0.70 – 0.91)
P	**2.0e-04**	**0.005**	**2.3e-05**	**9.3e-04**	**4.0e-04**	0.472	**5.2e-06**	**1.3e-04**	**0.001**
Intercept P	0.325	0.142	0.020	0.545	0.333	0.633	0.273	0.135	0.174

Abbreviations: MR = Mendelian Randomization; bioT = bioavailable testosterone; SHBG = sex hormone binding globulin, TT = total testosterone; CI = confidence interval; EstBB = Estonian Biobank; HUNT = HUNT Biobank; UFO = Umeå Fracture and Osteoporosis study; OR = odds ratio; OR and 95 % confidence intervals (CI) are given as OR estimates of one SD increase in bioavailable testosterone / SHBG / total testosterone; N total/cases = total number of women/women with fractures; N variants = number of genetic variants used; N var. excl. = Number of variants excluded in analysis.

Supplementary Table S4: Two sample Mendelian randomization results of the causal association between bioavailable testosterone, sex hormone binding globuline (SHBG) or total testosterone and bone mineral density (BMD).

**MR analysis type**	**Two-sample MR**			**Two-sample MR**			**Two-sample MR**		
**BMD**	FN-BMD			LS-BMD			FA-BMD		
**Cohort**	GEFOS meta-analyses, PMID 22504420			GEFOS meta-analyses, PMID 22504420			GEFOS meta-analyses, PMID 26367794		
**N**	22,177			22,990			7,848		
**Proportion of women**	100%			100%			96%*		
**Exposure**	bioT	SHBG	TT	bioT	SHBG	TT	bioT	SHBG	TT
**Primary MR analyses**									
**N variants**	145	279	207	144	279	205	166	341	234
**Inverse variance weighted MR**									
Beta (95% CI)	0.08 (0.02 – 0.14)	-0.08 (-0.16 – -0.004)	0.03 (-0.03 – 0.09)	0.13 (0.06 – 0.19)	-0.12 (-0.21 – -0.04)	0.08 (0.02 – 0.14)	0.24 (0.13 – 0.36)	-0.30 (-0.44 – -0.16)	0.11 (0.01 – 0.20)
P	**0.015**	**0.040**	0.316	**3.0e-04**	**0.004**	**0.009**	**2.40E-05**	**4.10E-05**	**0.024**
Q	177	348	237	200	391	254	265	434	333
Q P	**0.031**	**0.003**	0.069	**0.001**	**9.30E-06**	**0.01**	**1.30E-06**	**4.40E-04**	**1.90E-05**
I^2	18.7	20	13	28.5	28.8	19.6	37.7	21.6	29.9
**Weighted median MR**									
Beta (95% CI)	0.04 (-0.06 – 0.14)	-0.04 (-0.14 – 0.06)	0.06 (-0.03 – 0.15)	0.06 (-0.03 – 0.16)	-0.07 (-0.17 – 0.03)	0.14 (0.05 – 0.24)	0.13 (-0.03 – 0.30)	-0.13 (-0.34 – 0.08)	0.15 (0.01 – 0.28)
P	0.401	0.398	0.214	0.200	0.191	**0.003**	0.122	0.212	**0.035**
**Weighted mode MR**									
Beta (95% CI)	0.04 (-0.04 – 0.12)	-0.03 (-0.12 – 0.06)	0.06 (-0.08 – 0.21)	0.09 (0.00 – 0.17)	-0.09 (-0.18 – 0.003)	0.18 (0.02 – 0.34)	0.13 (-0.05 – 0.31)	-0.11 (-0.30 – 0.08)	0.27 (0.05 – 0.49)
P	0.285	0.491	0.400	**0.040**	0.061	**0.029**	0.172	0.245	**0.018**
**MR Egger**									
Beta (95% CI)	0.03 (-0.06 – 0.13)	-0.02 (-0.12 – 0.07)	0.05 (-0.08 – 0.18)	0.09 (-0.01 – 0.20)	-0.09 (-0.20 – 0.01)	0.09 (-0.05 – 0.23)	0.19 (-0.00 – 0.38)	-0.16 (-0.36 – 0.05)	0.17 (-0.00 – 0.35)
P	0.495	0.636	0.470	0.073	0.091	0.227	0.056	0.145	0.053
Intercept P	0.202	0.059	0.746	0.406	0.357	0.918	0.485	0.067	0.393
**MR PRESSO**									
Beta (95% CI)	0.08 (0.02 – 0.14)	-0.08 (-0.16 – -0.01)	0.03 (-0.03 – 0.09)	0.12 (0.05 – 0.18)	-0.12 (-0.21 – -0.04)	0.08 (0.02 – 0.14)	0.24 (0.13 – 0.36)	-0.30 (-0.44 – -0.16)	0.11 (0.01 – 0.20)
P	**0.016**	**0.030**	0.317	**6.1e-04**	**0.004**	**0.010**	**2.40E-05**	**4.10E-05**	**0.024**
N var. excl.	0	1	0	1	0	0	0	0	0
**MR LASSO**									
Beta (95% CI)	0.08 (0.02 – 0.13)	-0.07 (-0.14 – 0.002)	0.03 (-0.02 – 0.09)	0.13 (0.07 – 0.19)	-0.08 (-0.15 – -0.01)	0.09 (0.03 – 0.15)	0.22 (0.12 – 0.31)	-0.28 (-0.40 – -0.15)	0.05 (-0.04 – 0.14)
P	**0.009**	0.056	0.247	**2.3e-05**	**0.023**	**0.002**	**5.30E-06**	**2.8e-05**	0.26
N var. excl.	6	10	4	10	19	9	15	16	18
**MR RAPS**									
Beta (95% CI)	0.08 (0.01 – 0.14)	-0.07 (-0.15 – 0.01)	0.04 (-0.02 – 0.09)	0.12 (0.05 – 0.19)	-0.10 (-0.18 – -0.02)	0.09 (0.02 – 0.15)	0.24 (0.13 – 0.36)	-0.29 (-0.43 – -0.15)	0.10 (-0.00 – 0.19)
P	**0.016**	0.070	0.237	**9.9e-04**	**0.018**	**0.009**	**2.4e-05**	**7.1e-05**	0.055
**Steiger filtered analyses**									
**N variants**	135	256	195	134	249	195	116	248	172
**Inverse variance weighted MR**									
Beta (95% CI)	0.08 (0.03 – 0.13)	-0.07 (-0.14 – -0.01)	0.02 (-0.03 – 0.07)	0.12 (0.06 – 0.18)	-0.10 (-0.17 – -0.03)	0.07 (0.02 – 0.13)	0.14 (0.06 – 0.21)	-0.18 (-0.27 – -0.08)	0.09 (0.02 – 0.16)
P	**0.003**	**0.025**	0.395	**7.6e-05**	**0.006**	**0.011**	**7.00E-04**	**3.0e-04**	**0.011**
**Weighted median MR**									
Beta (95% CI)	0.04 (-0.05 – 0.14)	-0.04 (-0.15 – 0.06)	0.06 (-0.03 – 0.14)	0.06 (-0.03 – 0.16)	-0.07 (-0.17 – 0.04)	0.13 (0.04 – 0.23)	0.13 (-0.02 – 0.27)	-0.13 (-0.32 – 0.06)	0.13 (-0.00 – 0.27)
P	0.392	0.418	0.204	0.195	0.205	**0.004**	0.099	0.173	0.058
**Weighted mode MR**									
Beta (95% CI)	0.04 (-0.04 – 0.11)	-0.03 (-0.12 – 0.06)	0.07 (-0.08 – 0.22)	0.08 (-0.00 – 0.17)	-0.08 (-0.17 – 0.02)	0.19 (0.03 – 0.36)	0.08 (-0.00 – 0.17)	-0.08 (-0.17 – 0.02)	0.19 (0.03 – 0.36)
P	0.341	0.472	0.379	0.057	0.113	**0.024**	0.057	0.113	**0.024**
**MR Egger**									
Beta (95% CI)	0.03 (-0.05 – 0.12)	-0.03 (-0.12 – 0.06)	0.09 (-0.04 – 0.22)	0.10 (0.01 – 0.19)	-0.09 (-0.18 – 0.00)	0.06 (-0.07 – 0.19)	0.19 (0.03 – 0.35)	-0.15 (-0.34 – 0.04)	0.21 (0.05 – 0.36)
P	0.438	0.519	0.170	**0.024**	0.055	0.388	**0.019**	0.117	**0.009**
Intercept P	0.138	0.097	0.250	0.613	0.731	0.802	0.372	0.71	0.079

Abbreviations: MR = Mendelian Randomization; FN = Femoral neck; LS = Lumbar spine; FA = Forearm; bioT = bioavailable testosterone; SHBG = sex hormone binding globulin, TT = total testosterone; CI = confidence interval; Beta and 95 % confidence intervals (CI) are given as change in standard deviation (SD) of bone mineral density (BMD) per SD increase in bioT / SHBG / TT; N = total number of women; N variants = number of genetic variants used; N var. excl. = Number of variants excluded in analysis

* Calculated from supplementary table 18, PMID: 26367794

Supplementary Tables S5: Mendelian randomization results of (A) Forearm fractures and (B) BMD after removing genetic variants associated with potential confounders** at a P-value < 7×10^{-5}

A

**MR analysis type**	**One-sample MR**			**Two-sample MR**			**Mixed-sample MR**		
**Cohort**	UK Biobank			EstBB, HUNT, UFO			EstBB, HUNT, UFO, UK Biobank		
**Fracture**	Forearm			Forearm			Forearm		
**N total / cases**	237,572 / 11,564			111,351 / 8,823			348,923 / 20,387		
**Exposure**	bioT	SHBG	TT	bioT	SHBG	TT	bioT	SHBG	TT
**N variants before removal**	173	346	244	176	352	250	166	339	237
**N variants after removal**	136	285	193	132	278	193	123	267	180
**Inverse variance weighted MR**									
OR (95% CI)	0.72 (0.64 – 0.81)	1.77 (1.49 – 2.10)	0.79 (0.72 – 0.86)	0.76 (0.67 – 0.87)	1.68 (1.37 – 2.06)	0.89 (0.80 – 0.99)	0.74 (0.67 – 0.81)	1.75 (1.51 – 2.02)	0.83 (0.77 – 0.89)
P	**2.60E-08**	**9.90E-11**	**1.20E-07**	**1.00E-04**	**5.40E-07**	**0.034**	**1.80E-11**	**3.90E-14**	**6.70E-07**
Q	186	325	244	154	290	241	145	335	258
Q P	**2.00E-03**	**4.70E-02**	**6.00E-03**	0.086	0.289	**1.00E-02**	0.08	**2.00E-03**	**9.80E-05**
I^2	27.5	12.6	21.4	14.8	4.36	20.3	15.6	20.7	30.7
**Weighted median MR**									
OR (95% CI)	0.72 (0.62 – 0.85)	1.57 (1.20 – 2.06)	0.71 (0.62 – 0.80)	0.75 (0.60 – 0.94)	1.83 (1.31 – 2.56)	0.87 (0.72 – 1.05)	0.77 (0.67 – 0.87)	1.61 (1.30 – 1.99)	0.81 (0.73 – 0.89)
P	**5.60E-05**	**1.00E-03**	**8.60E-08**	**0.013**	**3.50E-04**	0.144	**7.40E-05**	**1.10E-05**	**3.00E-05**
**Weighted mode MR**									
OR (95% CI)	0.60 (0.39 – 0.92)	1.41 (0.97 – 2.06)	0.66 (0.56 – 0.78)	0.74 (0.54 – 1.03)	2.12 (1.32 – 3.40)	0.89 (0.75 – 1.06)	0.75 (0.59 – 0.95)	1.48 (1.10 – 1.99)	0.75 (0.66 – 0.84)
P	**0.02**	0.071	**1.20E-06**	0.073	**0.002**	0.205	**0.021**	**0.011**	**3.60E-06**
**MR Egger**									
OR (95% CI)	0.61 (0.47 – 0.79)	1.53 (1.08 – 2.17)	0.68 (0.58 – 0.79)	0.74 (0.55 – 0.99)	1.48 (0.99 – 2.19)	0.89 (0.74 – 1.07)	0.65 (0.54 – 0.79)	1.52 (1.15 – 2.03)	0.77 (0.67 – 0.87)
P	**3.00E-04**	**0.019**	**1.80E-06**	**0.047**	0.054	0.236	**3.00E-05**	**0.004**	**7.30E-05**
Intercept P	0.173	0.352	**0.021**	0.806	0.448	0.973	0.17	0.276	0.144

B

**MR analysis type**	**Two-sample MR**			**Two-sample MR**			**Two-sample MR**		
**BMD**	FN-BMD			LS-BMD			FA-BMD		
**Cohort**	GEFOS meta-analyses, PMID 22504420			GEFOS meta-analyses, PMID 22504420			GEFOS meta-analyses, PMID 26367794		
**N**	22,177			22,990			7,848		
**Proportion of women**	100%			100%			96%*		
**Exposure**	bioT	SHBG	TT	bioT	SHBG	TT	bioT	SHBG	TT
**N variants before removal**	145	279	207	144	279	205	166	341	234
**N variants after removal**	95	199	143	94	200	141	119	264	176
**Inverse variance weighted MR**									
Beta (95% CI)	0.07 (-0.03 – 0.16)	-0.19 (-0.34 – -0.04)	0.01 (-0.05 – 0.08)	0.14 (0.03 – 0.25)	-0.23 (-0.39 – -0.06)	0.07 (-0.01 – 0.14)	0.32 (0.18 – 0.46)	-0.45 (-0.65 – -0.25)	0.13 (0.03 – 0.23)
P	0.181	**0.014**	0.667	**0.012**	**0.006**	0.07	**7.40E-06**	**1.30E-05**	**0.01**
Q	99	257	151	126	287	181	169	326	224
Q P	0.343	**0.003**	0.284	**0.012**	**4.20E-05**	**0.011**	**2.00E-03**	**5.00E-03**	**7.00E-03**
I^2	5.02	23.1	6.05	26.4	30.7	22.7	30.1	19.3	21.9
**Weighted median MR**									
Beta (95% CI)	0.11 (-0.02 – 0.25)	-0.22 (-0.44 – -0.00)	0.05 (-0.05 – 0.15)	0.21 (0.06 – 0.35)	-0.13 (-0.35 – 0.09)	0.16 (0.05 – 0.27)	0.27 (0.08 – 0.46)	-0.31 (-0.63 – 0.01)	0.16 (0.01 – 0.31)
P	0.087	0.048	0.319	**0.004**	0.235	**0.004**	**0.005**	0.061	**0.035**
**Weighted mode MR**									
Beta (95% CI)	0.15 (-0.10 – 0.40)	-0.14 (-0.47 – 0.18)	0.06 (-0.08 – 0.20)	0.21 (-0.08 – 0.50)	-0.17 (-0.56 – 0.22)	0.19 (0.00 – 0.37)	0.48 (0.04 – 0.93)	-0.19 (-0.66 – 0.29)	0.28 (0.07 – 0.50)
P	0.244	0.398	0.412	**0.16**	0.393	**0.047**	**0.037**	0.441	**0.01**
**MR Egger**									
Beta (95% CI)	0.05 (-0.23 – 0.33)	-0.02 (-0.35 – 0.32)	0.05 (-0.09 – 0.19)	0.46 (0.14 – 0.78)	-0.28 (-0.64 – 0.09)	0.08 (-0.08 – 0.24)	0.41 (0.12 – 0.71)	-0.50 (-0.89 – -0.11)	0.20 (0.03 – 0.38)
P	0.738	0.928	0.470	**0.006**	0.141	0.314	**0.007**	**0.013**	**0.025**
Intercept P	0.901	0.254	0.554	**0.042**	0.77	0.836	0.477	0.779	0.344

Abbreviations: MR = Mendelian Randomization; bioT = bioavailable testosterone; SHBG = sex hormone binding globulin, TT = total testosterone; CI = confidence interval; Beta and 95 % confidence intervals (CI) are given as OR estimates (fractures) or change in standard deviation (SD) of bone mineral density (BMD) per SD increase in bioT / SHBG / TT; EstBB = Estonian Biobank; HUNT = HUNT Biobank; UFO = Umeå Fracture and Osteoporosis study, N = total number of women; N variants = number of genetic variants;

* Calculated from supplementary table 18, PMID: 26367794

**See Supplementary Table S6 for details: BMI, estradiol, calcium levels, physical activity, grip strength, rheumatoid arthritis, glucocorticoid use, smoking status, alcohol intake, educational attainment, household income

Supplementary Table S6: Details of GWAS used to identify genetic variants associated with potential confounders for forearm fractures and bone mineral density (see Supplementary Tables S5). All GWAS data were sourced from the MRC IEU OpenGWAS infrastructure*.

GWAS ID	Trait	N	Sex	Population	N SNPs P < $7x10^5$
ieu-b-40	Body Mass Index	681,275	Males and Females	European	27
ieu-b-4872	Oestradiol	53,391	Females	European	6
ebi-a-GCST90025990	Calcium levels	400,792	Males and Females	European	27
ebi-a-GCST006099	Accelerometer-based physical activity measurement	91,084	Males and Females	European	0
ukb-a-508	Number of days/week of moderate physical activity 10+ minutes	321,309	Males and Females	European	3
ukb-b-151	Number of days/week of vigorous physical activity 10+ minutes	440,512	Males and Females	European	2
ebi-a-GCST006097	Moderate to vigorous physical activity levels	377,234	Males and Females	European	2
ukb-b-10215	Hand grip strength (right)	461,089	Males and Females	European	25
ukb-b-7478	Hand grip strength (left)	461,026	Males and Females	European	16
ebi-a-GCST90018910	Rheumatoid arthritis	417,256	Males and Females	European	1
ebi-a-GCST90019000	Medication use (glucocorticoids)	205,700	Males and Females	European	3
ebi-a-GCST90029013	Educational attainment (years of education)	461,457	Males and Females	European	15
ukb-b-7408	Average total household income before tax	397,751	Males and Females	European	4
ukb-a-225	Smoking status: Current	336,024	Males and Females	European	4
ebi-a-GCST90029014	Smoking status	468,170	Males and Females	European	6
ieu-b-4834	Alcohol consumption	83,626	Males and Females	European	1
ukb-b-5779	Alcohol intake frequency	462,346	Males and Females	European	23

Abbreviations: GWAS = Genome Wide Association Studies; GWAS ID = ID of GWAS in the MRC IEU OpenGWAS database; N = total sample size; SNP = single-nucleotide polymorphism; N SNPs P < 6×10^{-5} = Number of SNPs among GWAS significant bioavailable testosterone / sex hormone binding globulin / total testosterone SNPs that are associated with the trait at a P-value level < 7×10^{-5}

* https://gwas.mrcieu.ac.uk

Supplementary Table S7: Results from Mendelian randomization analyses of three individual SHBG cis-SNPs (Wald ratios) and combined analyses with all three SHBG cis-SNPs (IVW MR) for (A) forearm fractures and (B) bone mineral density (BMD)

A

**MR analysis type**	**One-sample MR**		**Two-sample MR**		**Mixed-sample MR**	
**Cohort**	UK Biobank		EstBB, HUNT, UFO		EstBB, HUNT, UFO, UK Biobank	
**Fracture**	Forearm		Forearm		Forearm	
**N total / cases**	237,572 / 11,564		111,351 / 8,823		348,923 / 20,387	
**Exposure**	SHBG		SHBG		SHBG	
**Wald ratios**	OR (95% CI)	pval	OR (95% CI)	pval	OR (95% CI)	pval
rs1799941	1.70 (1.23 – 2.34)	**0.001**	1.76 (1.20 – 2.58)	**0.004**	1.72 (1.35 – 2.21)	**1.50E-05**
rs6761	1.15 (0.66 – 2.01)	0.613	2.70 (1.40 – 5.20)	**0.003**	1.65 (1.08 – 2.52)	**0.021**
rs858519	1.33 (1.00 – 1.77)	0.051	1.53 (1.08 – 2.16)	**0.016**	1.41 (1.13 – 1.75)	**0.002**
**IVW MR**	1.47 (1.13 – 1.90)	**0.004**	1.59 (1.16 – 2.16)	**0.003**	1.51 (1.24 – 1.85)	**4.20E-05**

B

**MR analysis type**	**Two-sample MR**		**Two-sample MR**		**Two-sample MR**	
**BMD**	FN-BMD		LS-BMD		FA-BMD	
**Cohort**	GEFOS meta-analyses, PMID 22504420		GEFOS meta-analyses, PMID 22504420		GEFOS meta-analyses, PMID 26367794	
**N**	22,177		22,990		7,848	
**Proportion of women**	100%		100%		96%*	
**Exposure**	SHBG		SHBG		SHBG	
**Wald ratios**	Beta (95% CI)	pval	Beta (95% CI)	pval	Beta (95% CI)	pval
rs1799941**	-0.24 (-0.47 – 0.00)	0.050	-0.07 (-0.31 – 0.17)	0.591	-0.43 (-0.77 – -0.09)	**0.014**
rs6761	-0.24 (-0.59 – 0.12)	0.192	-0.19 (-0.55 – 0.18)	0.311	-0.18 (-0.78 – 0.42)	0.553
rs858519**	0.02 (-0.17 – 0.20)	0.857	-0.10 (-0.28 – 0.09)	0.315	-0.13 (-0.45 – 0.18)	0.395
**IVW MR**	-0.05 (-0.22 – 0.13)	0.597	-0.08 (-0.26 – 0.09)	0.348	-0.25 (-0.53 – 0.03)	0.074

Abbreviations: MR = Mendelian Randomization; SHBG = sex hormone binding globulin, CI = confidence interval; Beta and 95 % confidence intervals (CI) are given as change in standard deviation (SD) of bone mineral density (BMD) per SD increase in SHBG; EstBB = Estonian Biobank; HUNT = HUNT Biobank; UFO = Umeå Fracture and Osteoporosis study; FN = Femoral neck; LS = Lumbar spine; FA = Forearm; N = total number of women; IVW MR = Inverse variance weighted MR accounting for correlation among genetic variants

* Calculated from supplementary table 18, PMID: 26367794
** The LS-BMD and FN-BMD datasets were restricted to HapMap variants, with rs6761 being the only available cis-SNP. For the remaining two cis-SNPs, we employed SNPs: rs12150660 as a substitute for rs1799941, and rs727428 was used in place of rs858519.

Supplementary Figure S1: Flow chart of study methodology.

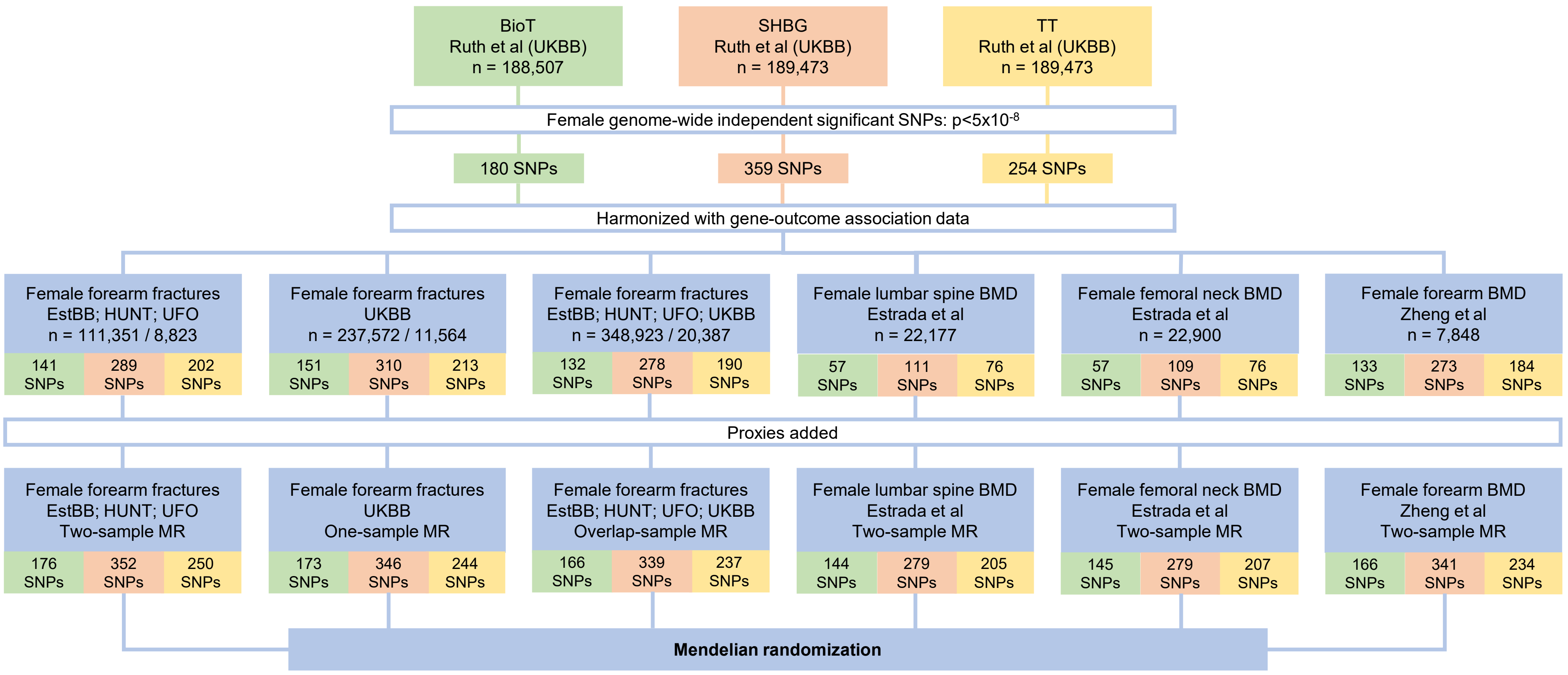

Abbreviations: SNP = single-nucleotide polymorphisms; BioT = bioavailable testosterone; SHBG = sex hormone binding globulin, TT = total testosterone; BMD = bone mineral density; EstBB = Estonian Biobank; HUNT = HUNT Biobank; UFO = Umeå Fracture and Osteoporosis study; UKBB = UK Biobank; n = total number of women / total number of cases;

Supplementary Figure S2: Power (%) to detect a causal association (y-axis) given the size of the true underlying causal effect of one standard unit increase in sex hormone binding globulin (SHBG, N = 189,473), bioavailable testosterone (BioT, N = 188,507) or total testosterone (TT, N = 230,454) on forearm fracture risk (x-axis; odds ratio, OR; standard deviation, SD), using two-sample MR fracture cases, N = 111,351 (cases = 8,823). We estimate that the 359 SNPs associated with SHBG explain 17,6% of the variance of SHBG, 180 SNPs associated with BioT explain 7.0% of the variance of BioT and the 254 SNPs associated with TT explain 9.3% of the variance of TT.

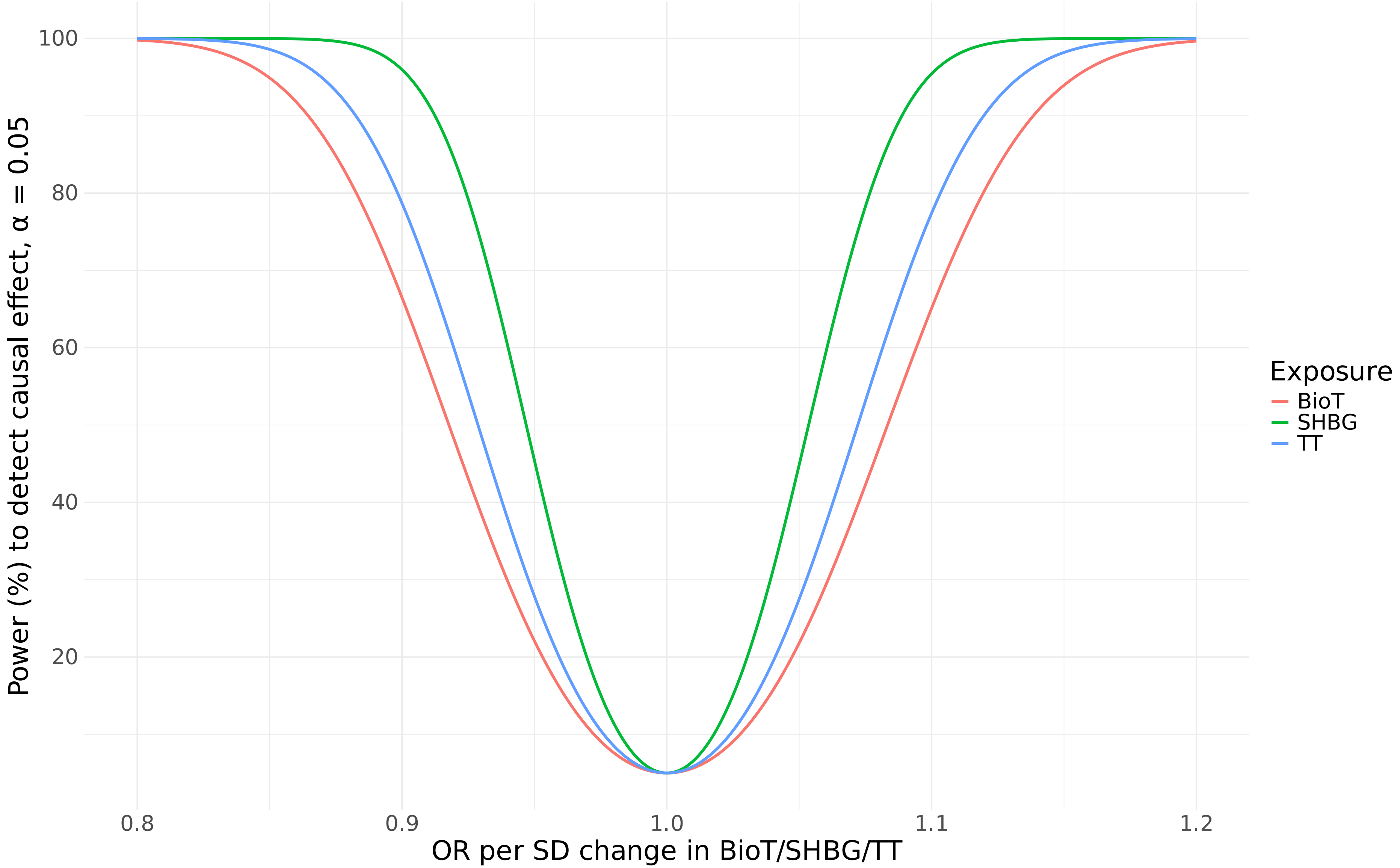

Supplementary Figure S3: Scatter plots of associations in women between exposures (x-axis) - (a) bioavailable testosterone (BioT), (b) sex hormone binding globulin (SHBG), (c) total testosterone (TT) - and outcomes (y-axis) - (1) forearm fractures (EstBB,HUNT,UFO), (2) forearm BMD (FA-BMD), (3) lumbar spine BMD (LS-BMD), (4) femoral neck BMD (FN-BMD) - with standard error bars. The slopes of the colored lines correspond to the estimated causal effect obtained with each method used.

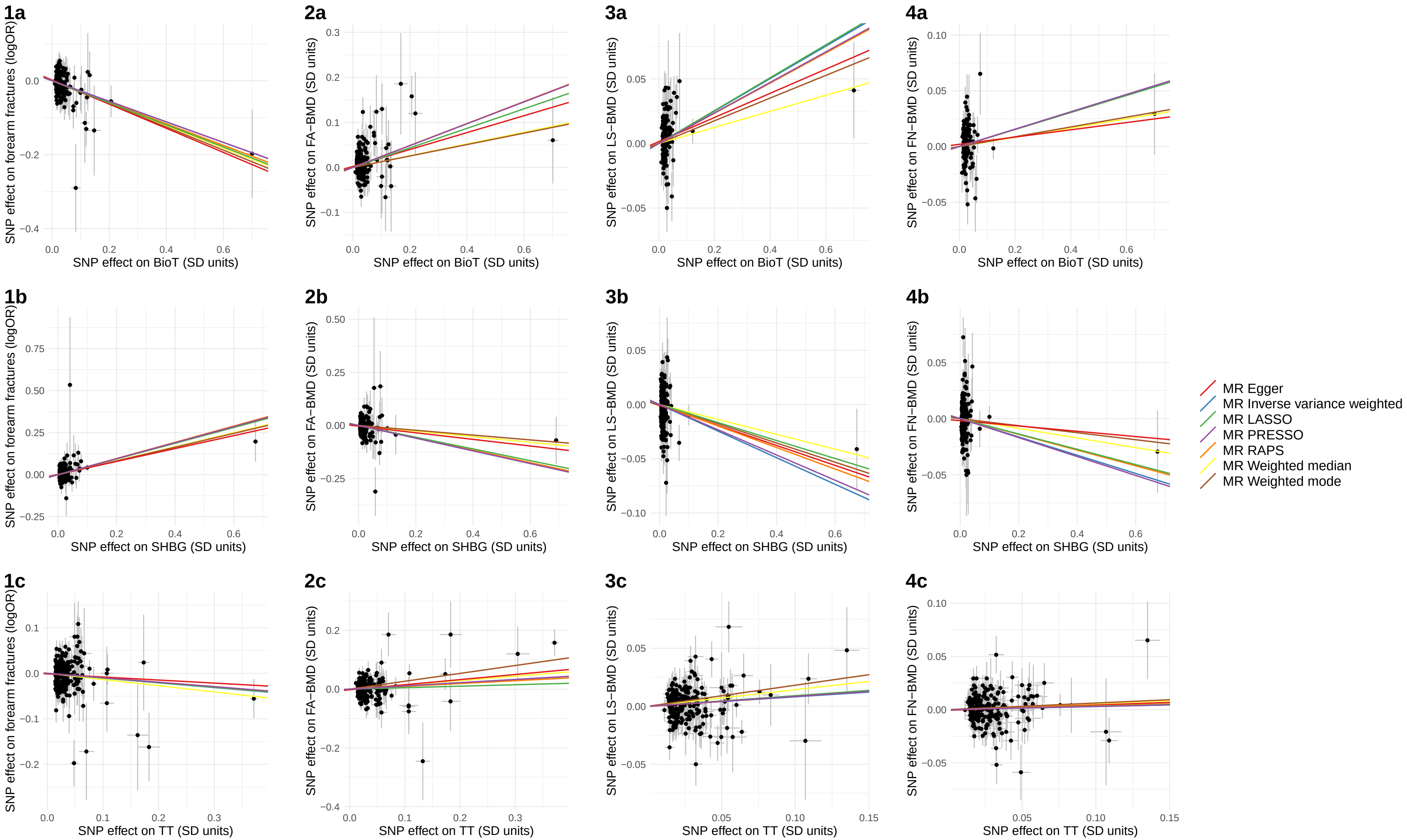

Abbreviations: BMD = bone mineral density; MR = Mendelian Randomization; SNP = single-nucleotide polymorphisms; OR = odds ratio; SD = standard deviation, EstBB = Estonian Biobank; HUNT = HUNT Biobank; UFO = Umeå Fracture and Osteoporosis study

Supplementary Figure S4: Funnel plot showing the relationship between the causal effect of the exposures - (a) bioavailable testosterone (BioT), (b) sex hormone binding globulin (SHBG), (c) total testosterone (TT) - on the outcome - (1) forearm fractures (EstBB, HUNT,UFO), (2) forearm BMD (FA-BMD), (3) lumbar spine BMD (LS-BMD), (4) femoral neck BMD (FN-BMD) - estimated using the Wald ratio estimate for each SNP (x-axis) against the inverse of the standard error of such estimate (y-axis). Vertical lines show the causal estimates using all SNPs combined into a single instrument for each different MR method.

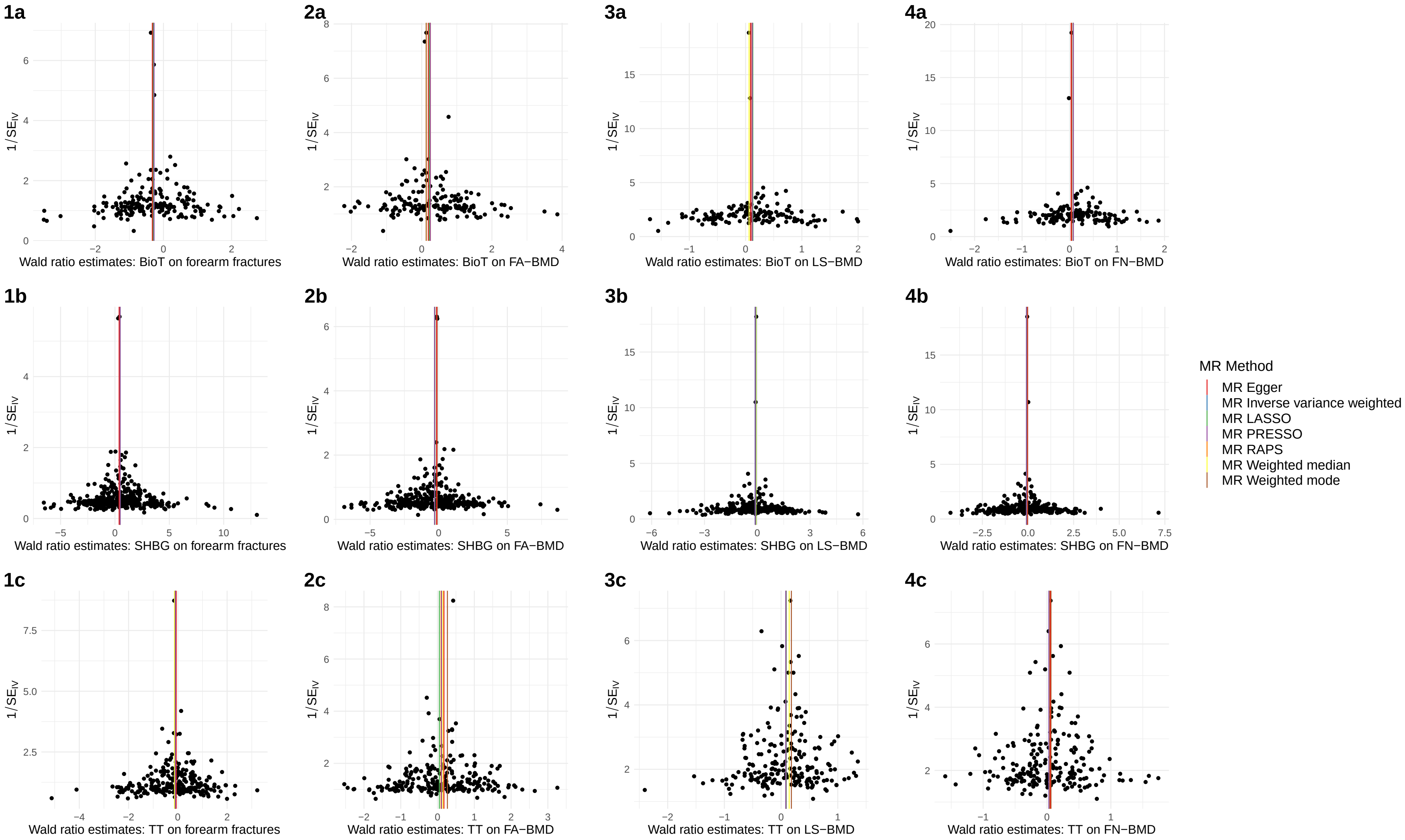

Abbreviations: BMD = bone mineral density; MR = Mendelian Randomization; SNP = single-nucleotide polymorphisms; EstBB = Estonian Biobank; HUNT = HUNT Biobank; UFO = Umeå Fracture and Osteoporosis study
